# Supplementary material for: A neurodegeneration checkpoint mediated by REST protects against the onset of Alzheimer’s disease
Source: Nat Commun. 2023 Nov 2;14:7030. doi: 10.1038/s41467-023-42704-6 (PMC10622455; doi:10.1038/s41467-023-42704-6)

A Neurodegeneration Checkpoint Mediated by REST Protects Against the Onset of Alzheimer's Disease

Liviu Aron, Chenxi Qiu, Zhen Kai Ngian, Marianna Liang, Derek Drake, Jaejoon Choi, Marty A. Fernandez, Perle Roche, Emma L. Bunting, Ella K. Lacey, Sara E. Hamplova, Monlan Yuan, Michael S. Wolfe, David A. Bennett, Eunjung A. Lee, and Bruce A. Yankner

This file contains Supplementary Figures 1-11

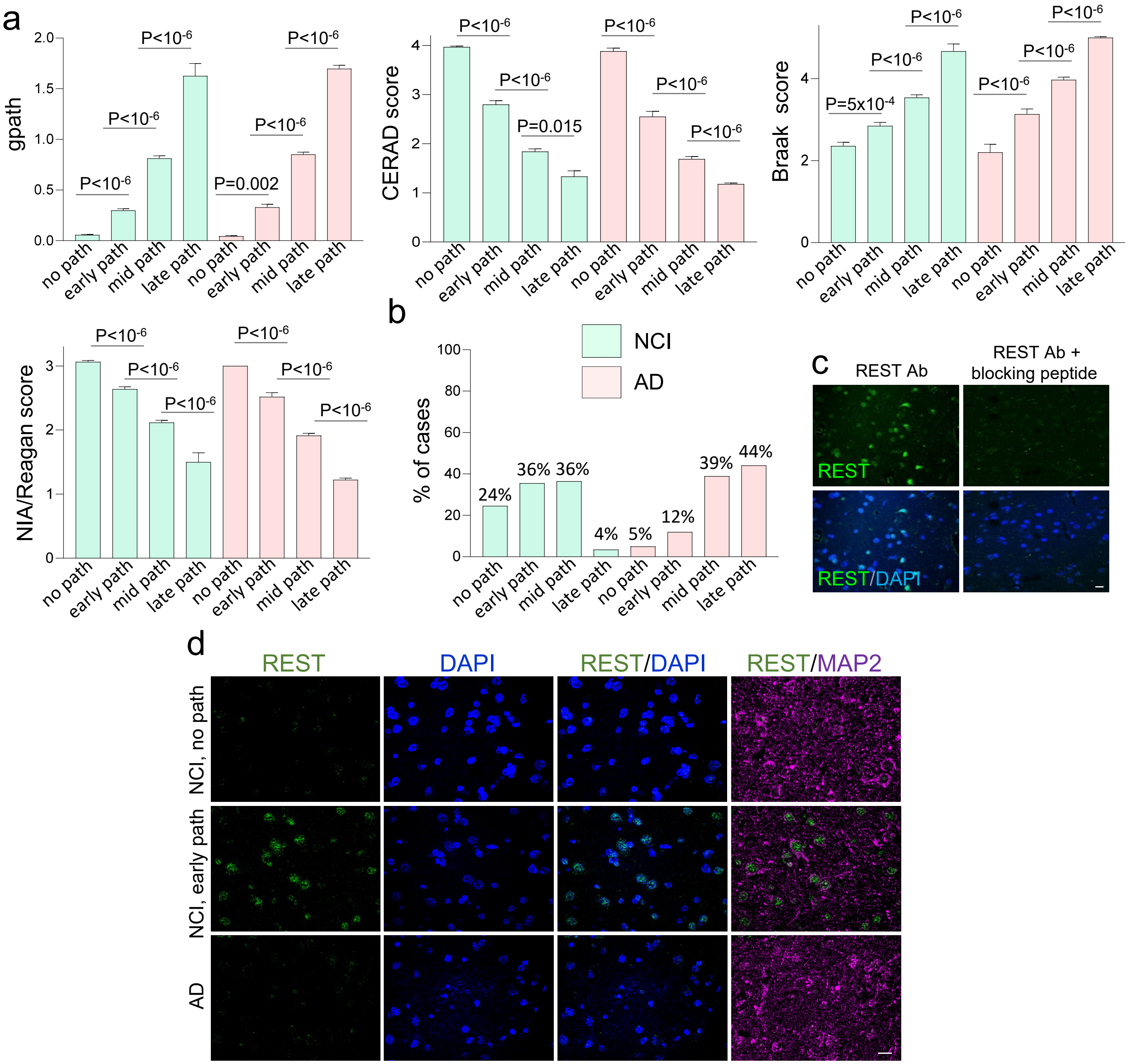

Supplementary Figure 1. Induction of REST in association with early AD pathology. a Partitioning of the aging population based on cognitive status (NCI or AD) and levels of AD pathology (no pathology, early, mid or late AD pathology). A representative sample of age-matched cases with no cognitive impairment (NCI, n=518 cases) and AD (n=501 cases) from the ROSMAP cohort were partitioned based on indices of AD pathology (gpath: composite measure of Aβ neuritic and diffuse plaque load and neurofibrillary tangle densities across 5 brain regions; CERAD score: a measure of total plaque load; Braak score: a measure of neurofibrillary tangle load; and NIA/Reagan score: a composite measure of plaque and neurofibrillary tangle load). Note that the differences in the indices of AD pathology between groups within each sample (NCI or AD) are highly significant. Adjusted P-values generated by two-way ANOVA with Tukey's post-hoc test are shown. **b** Population distribution of NCI and AD cases with no pathology, early pathology, mid pathology and late pathology (NCI, n=518; AD, n=501). Values represent the mean ± S.E.M. (**a**) or the mean (**b**). **c** Specificity of REST immunolabeling. Labeling of prefrontal cortex with an antibody against the REST C-terminal domain (green, Bethyl IHC-00141) and DAPI shows nuclear REST immunoreactivity in an NCI case with early AD pathology. Preincubation of the antibody with a REST C-terminal peptide (REST residues 1000-1097) abolishes immunoreactivity. **d** Immunolabeling of REST (green), the neuron marker MAP2 (magenta) and DNA (blue) in NCI cases with no pathology or early AD pathology, as well as AD shows low REST expression in NCI cases with no AD pathology, increased REST nuclear expression in neurons of the prefrontal cortex in NCI cases with early AD pathology and loss of REST in AD. Scale bars, 25 μm. Source data are provided as a Source Data file.

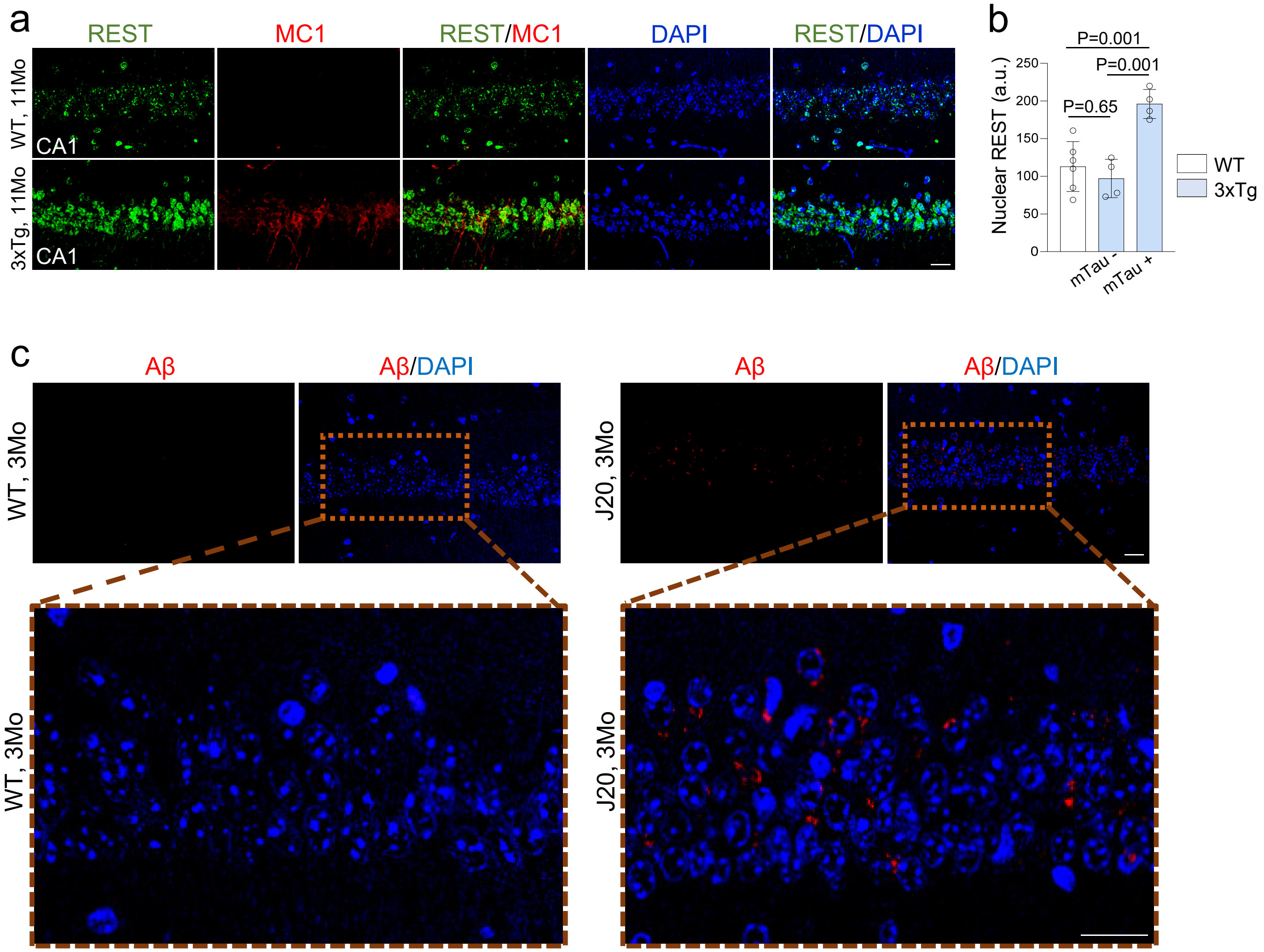

Supplementary Figure 2. Induction of REST in mice with early AD pathology. a,**b** REST induction in neurons with misfolded tau in 3xTg mice. **a** Immunolabeling of REST (green) and a pathologic, conformationally-altered epitope of tau (antibody MC1, red; mTau) in the CA1 sector of the hippocampus shows increased nuclear REST in neurons that accumulate mTau in 11-month-old 3xTg mice. **b** Quantification of nuclear REST levels in hippocampal CA1 neurons in 11-month-old WT (n=6) and 3xTg (n=4) mice. Shown are values for neurons in WT mice, and for MC1-positive (mTau+) or MC1-negative (mTau-) neurons in 3xTg mice. Shown are individual values and the mean ± S.E.M, as well as adjusted P-values generated by one-way ANOVA with Tukey's post-hoc test. **c** Immunolabeling of human Aβ (red) and DNA (blue) in the hippocampus CA1 region of 3-month-old WT and J-20 mice shows cellular accumulation of human Aβ in J20 but not WT mice. No amyloid plaques are present at this stage. Scale bars, 25 μm. Source data are provided as a Source Data file.

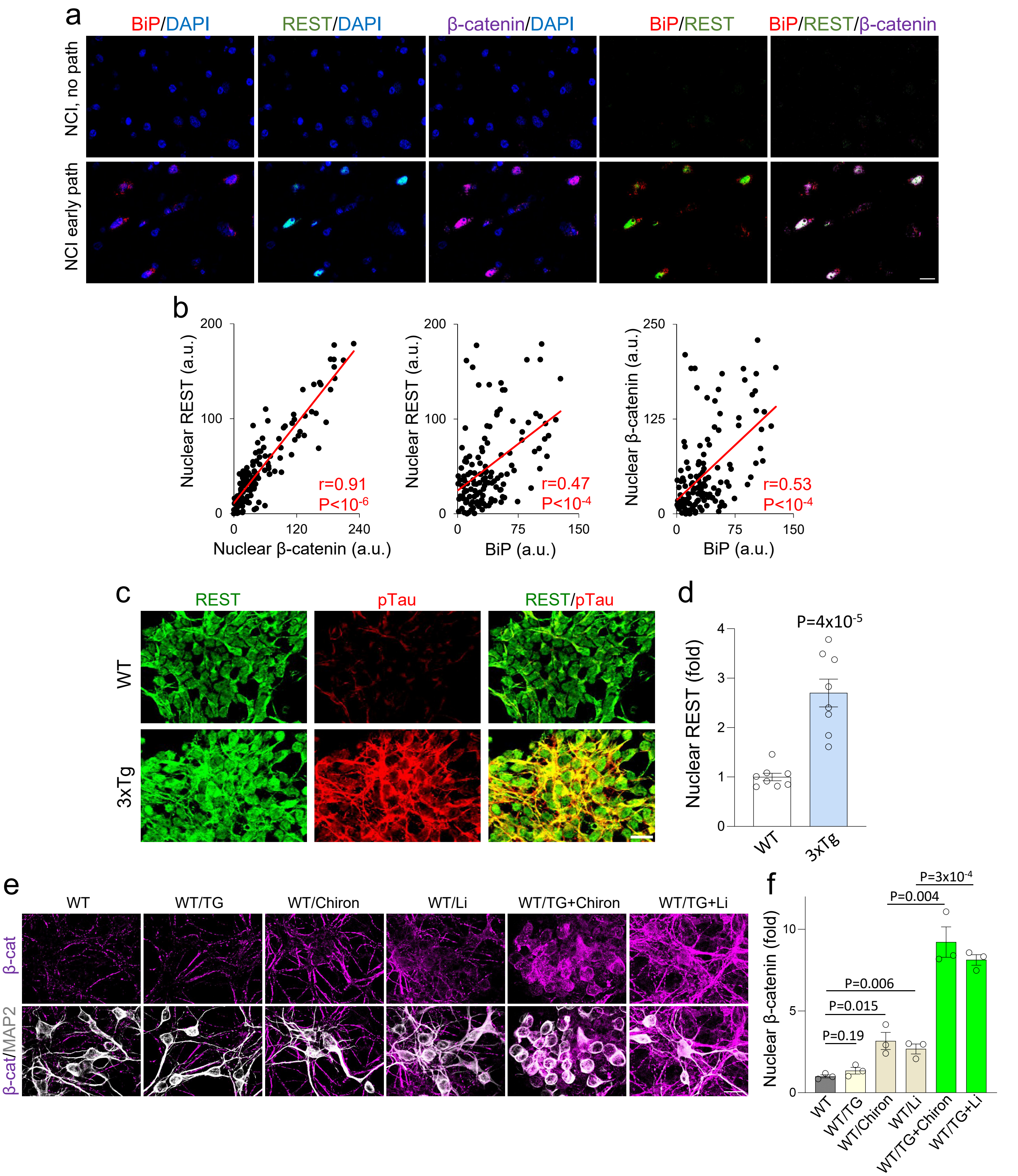

Supplementary Figure 3. Role of the UPR and β-catenin signaling in REST activation. a Immunolabeling of UPR activation (marker BiP/GRP78, red), REST (green), β-catenin (magenta) and DNA (DAPI) in an NCI case with no AD pathology (upper panel) and an NCI case with early AD pathology (lower panel) shows coordinate upregulation of BiP, nuclear REST and nuclear β-catenin expression in the NCI case with early AD pathology. **b** Correlation between nuclear β-catenin and nuclear REST (left graph), BiP and nuclear REST (middle graph) and

BiP and nuclear β-catenin (right graph) levels in the prefrontal cortex of n=3 NCI cases with early AD pathology. Shown are the mean fluorescence intensity values for nuclear REST in individual cortical neurons. a.u.- arbitrary units. The Pearson r correlation coefficients and P-values are shown. **c,d** REST induction in 3xTg cortical neurons in culture. **c** Immunolabeling for REST (green) and pSer202-tau (CP13 antibody, red; pTau) in WT and 3xTg primary cortical neurons after 11 days in vitro (DIV11). **d** Quantification of nuclear REST levels (n=8 independent experiments). **e** Immunolabeling of β-catenin (magenta) and neuron marker MAP2 (white) in WT primary cortical neurons (PCNs) shows low nuclear β-catenin in WT neurons treated with vehicle (CTRL) or the UPR inducer thapsigargin (TG) for 24 hours, mildly increased nuclear β-catenin expression after treatment with the GSK3β inhibitors CHIR99021 (Chiron) and lithium chloride (Li), and strongly increased nuclear β-catenin levels after treatment with a combination of TG and Chiron, or a combination of TG and Li. **f** Quantification of average nuclear β-catenin levels, expressed as mean fluorescence intensity/nucleus (in arbitrary units, a.u.) for n=3 independent experiments. **d,f** Shown are the means ± S.E.M. as well as each individual biological replicate. P-values generated by two-tailed unpaired t-tests are shown in (**d**) and (**f**) (planned comparisons). Scale bars, 25 µm. Source data are provided as a Source Data file.

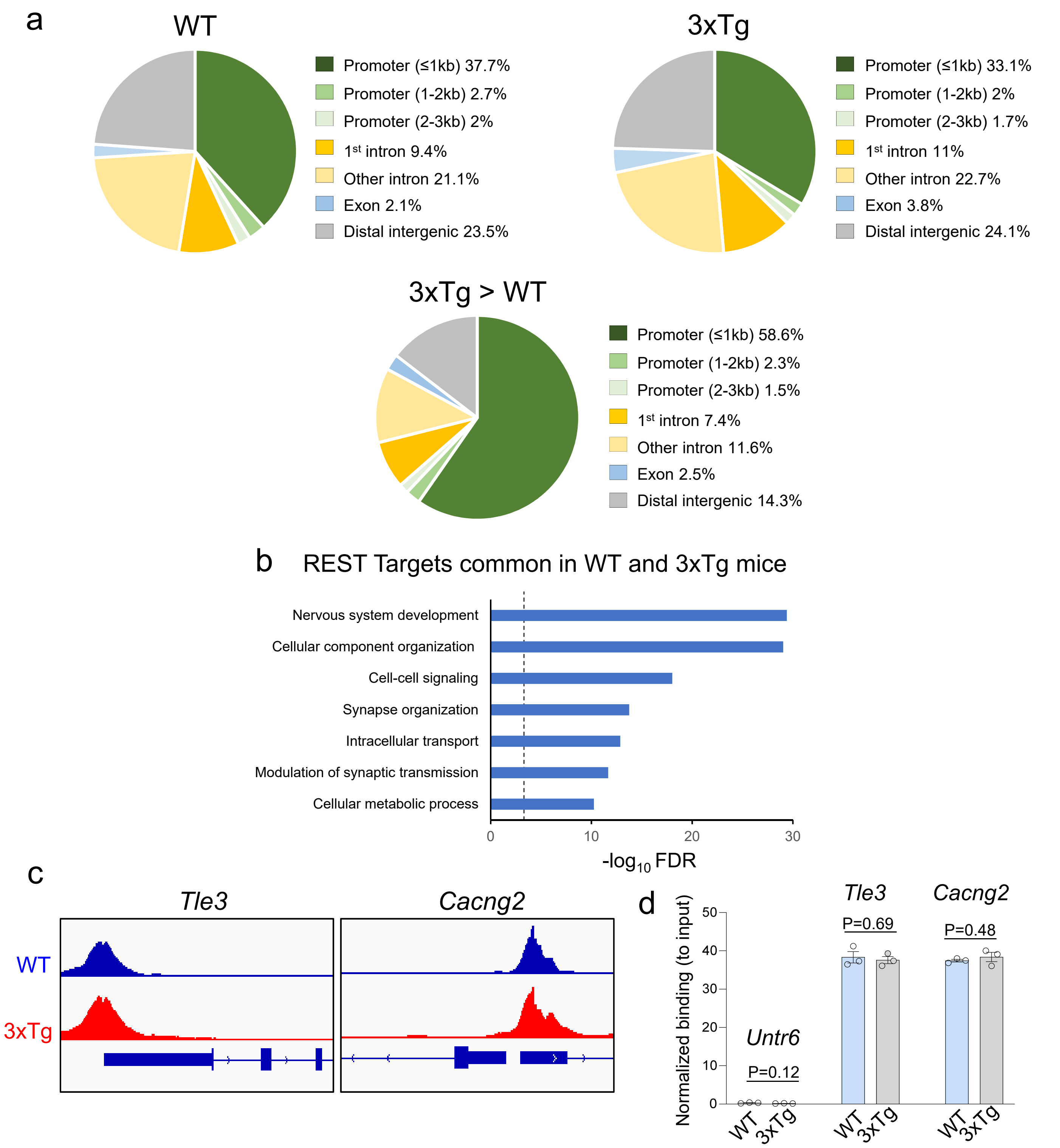

Supplementary Figure 4. REST ChIP-seq analysis. a Genomic distribution of REST binding in WT and 3xTg mice, as well as the regions significantly enriched in 3xTg mice (3xTg > WT). **b** Gene ontology (GO) term analysis of REST gene targets that are shared between WT and 3xTg mice. **c** Examples of REST binding to Tle3, and Cacng2 genes in WT (blue: average of 4 biological replicates) and 3xTg (red: average of 4 biological replicates) mouse cortex resolved by ChIP-seq. **d** ChIP-seq results were validated by qPCR amplification of ChIP DNA using primers for 2 gene loci associated with ChIP-seq peaks (Tle3, and Cacng2), and a locus with no detectable REST binding on chromosome 6 (Untr6). Shown are individual binding values normalized to the input control and the mean ± S.E.M. P-values generated by two-tailed unpaired t-tests are shown. Source data are provided as a Source Data file.

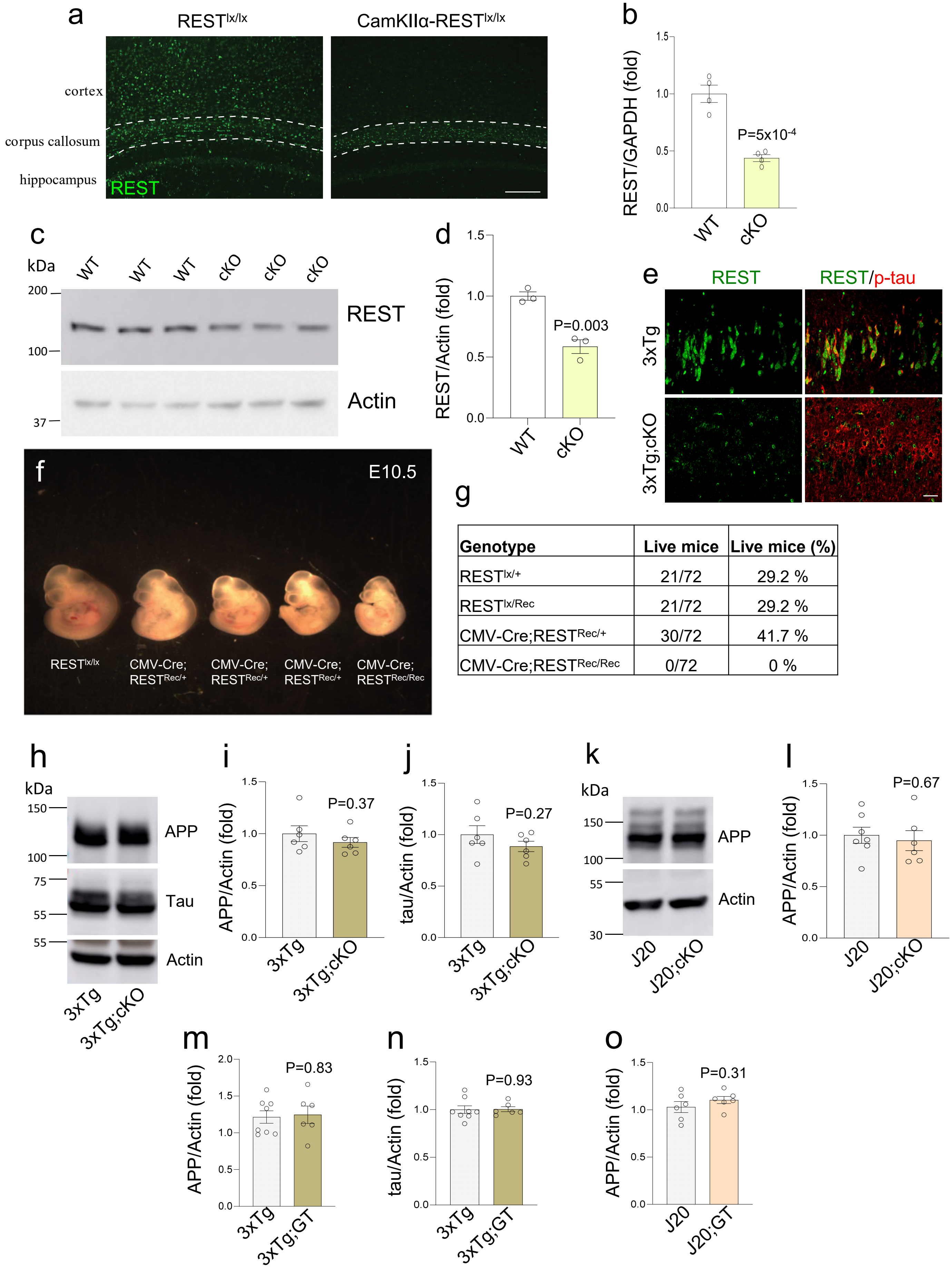
a
REST lx/lx
CamKIIα-REST lx/lx
cortex
corpus callosum
hippocampus
REST
b
REST/GAPDH (fold)
P=5x10-4
WT
cKO
c
kDa
WT
WT
WT
cKO
cKO
cKO
200
100
37
REST
Actin
d
REST/Actin (fold)
P=0.003
WT
cKO
e
REST
REST/p-tau
3xTg
3xTg;cKO
f
E10.5
REST lx/lx
CMV-Cre; REST Rec/+
CMV-Cre; REST Rec/+
CMV-Cre; REST Rec/+
CMV-Cre; REST Rec/Rec
g
Genotype | Live mice | Live mice (%)
REST lx/+ | 21/72 | 29.2 %
REST lx/Rec | 21/72 | 29.2 %
CMV-Cre;REST Rec/+ | 30/72 | 41.7 %
CMV-Cre;REST Rec/Rec | 0/72 | 0 %
h
kDa
150
100
75
55
55
APP
Tau
Actin
3xTg
3xTg;cKO
i
APP/Actin (fold)
P=0.37
3xTg
3xTg;cKO
j
tau/Actin (fold)
P=0.27
3xTg
3xTg;cKO
k
kDa
150
100
55
30
APP
Actin
J20
J20;cKO
l
APP/Actin (fold)
P=0.67
J20
J20;cKO
m
APP/Actin (fold)
P=0.83
3xTg
3xTg;GT
n
tau/Actin (fold)
P=0.93
3xTg
3xTg;GT
o
APP/Actin (fold)
P=0.31
J20
J20;GT

Supplementary Figure 5. Conditional and genetrap REST loss-of-function alleles.

a REST immunolabeling (green) of adult WT and forebrain-wide glutamatergic neuron-specific CamKIIα-Cre;RESTlx/lx mice showing loss of REST expression in most cortical and hippocampal neurons, but no loss of oligodendrocytic REST (in the white matter/corpus callosum). Scale bar, 150 µm. **b** qRT-PCR analysis of total mRNA extracted from the WT and CamKIIα-Cre;$REST^{lx/lx}$ (cKO) cortex shows partial loss of REST mRNA in REST cKO mice. Note that REST is still expressed in glia. **c** Western blot analysis of REST protein and actin in WT and CamKIIα-Cre;$REST^{lx/lx}$ (cKO) cortex. **d** Quantification of REST relative to actin. n=3 mice/group. **e** Immunolabeling for REST (green) and pSer202 tau (antibody CP13; red) in 12-month-old 3xTg and 3xTg;cKO hippocampus shows loss of REST expression in CA1 neurons in 3xTg;cKO mice. Scale bars, 25 µm. **f** Brightfield micrograph of E10.5 embryos of the indicated genotypes. **g** Summary of the numbers and frequencies (%) of viable progeny from CMV-Cre;$REST^{rec/+}$ x $REST^{lx/lx}$ crosses. Viable mice were determined 3 weeks after birth. We found no viable CMV-Cre;$REST^{rec/rec}$ animals. $REST^{rec}$ indicates the Cre-recombined $REST^{lx}$ allele. **h-j** Western blot detection (**h**) and quantification (**i**,**j**) of human APP (**i**) and tau (**j**) in the hippocampus of 6-month-old 3xTg and 3xTg;cKO mice (n=6 mice/group). **k**,**l** Western blot detection (**k**) and quantification (**l**) of human APP in the hippocampus of 6 month old J-20 and J-20;cKO mice (n=7 mice/group). Actin was used as loading control. **m,n** Quantification of human APP (**m**) and tau (**n**) levels by Western blot analysis of the hippocampus of 28-29-month-old 3xTg (n=8) and 3xTg;GT (n=6) mice. **o** Quantification of human APP levels by Western blot analysis of the hippocampus of 28-29-month-old J-20 and J-20;GT mice (n=6 mice/group). REST deletion in 3xTg and J-20 mice did not significantly alter human APP or tau mRNA levels (data not shown). **b**,**d**,**i**,**j**,**l**,**m**,**n**,**o** Shown are individual values, the mean ± S.E.M, as well as P-values generated by two-tailed unpaired t-test. Source data are provided as a Source Data file.

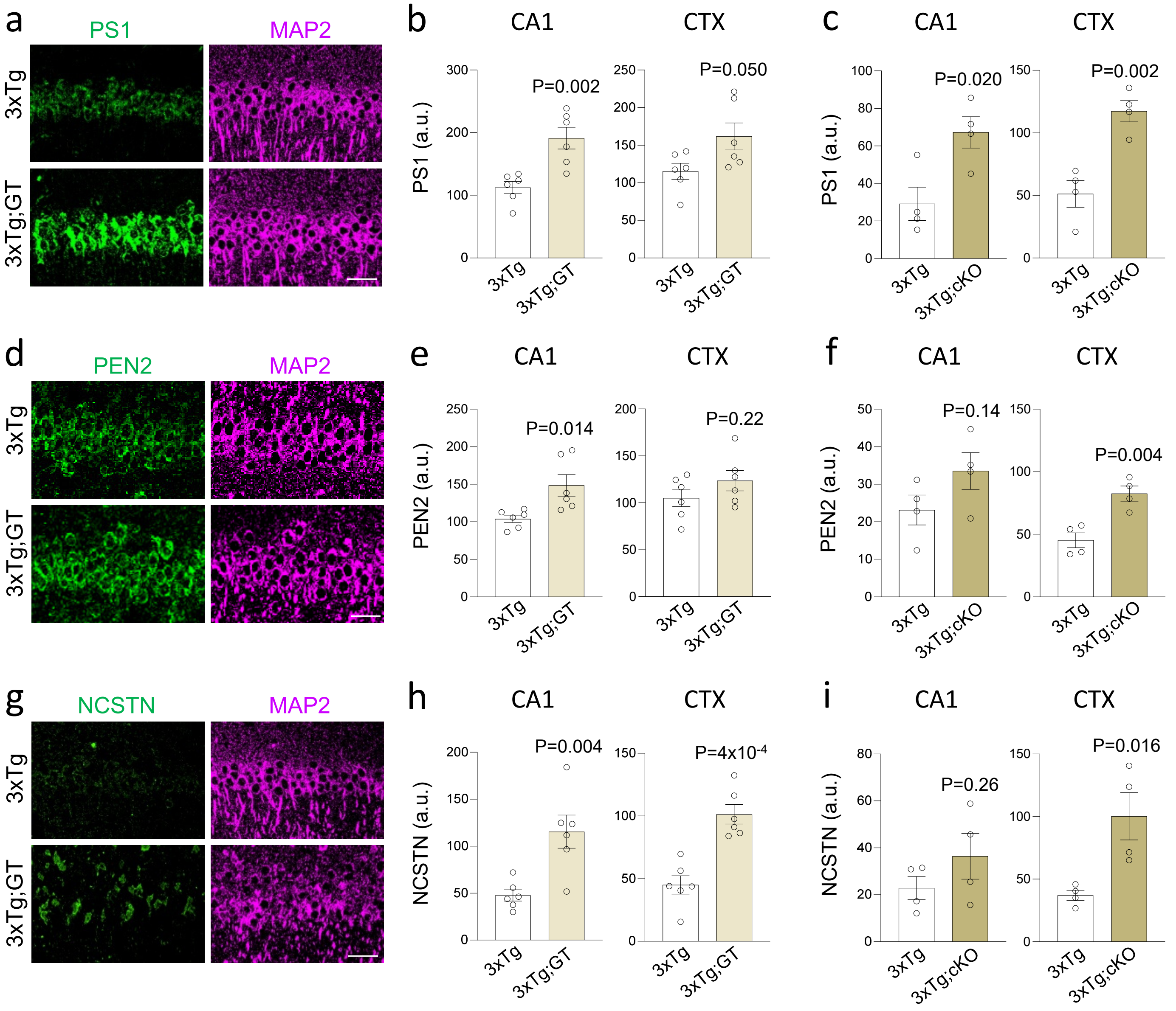

Supplementary Figure 6. REST inhibits expression of γ-secretase components in the brain. a,b Partial REST inactivation increases neuronal PS1 expression. **a** Immunolabeling for presenilin 1 (PS1; green) and MAP2 (magenta) in the CA1 sector of the hippocampus in 29-month-old 3xTg and 3xTg;GT mice. **b** Quantification of PS1 immunofluorescence intensity in hippocampal CA1 and the cortex of 28-29-month-old 3xTg (n=6) and 3xTg;GT (n=6) mice. **c** Quantification of PS1 immunofluorescence intensity in hippocampal CA1 and the cortex of 9-month-old 3xTg (n=4) and 3xTg;cKO (n=4) mice. **d,e** Partial REST inactivation increases neuronal PEN-2 expression. **d** Immunolabeling for PEN2 (green) and the neuronal marker MAP2 (magenta) in the CA1 sector of the hippocampus in 29-month-old 3xTg and 3xTg;GT mice. **e** Quantification of PEN2 immunofluorescence intensity in hippocampal CA1 and the cortex of 28-29-month-old 3xTg (n=6) and 3xTg;GT (n=6) mice. **f** Quantification of PEN2 immunofluorescence intensity in hippocampal CA1 and the cortex of younger 9-month-old 3xTg (n=4) and 3xTg;cKO (n=4) mice. **g,h** Partial REST inactivation increases neuronal nicastrin expression. **g** Immunolabeling for nicastrin (NCSTN; green) and MAP2 (magenta) in the CA1 sector of the hippocampus in 29-month-old 3xTg and 3xTg;GT mice. **h** Quantification of NCSTN immunofluorescence in hippocampal CA1 and the cortex of 28-29-month-old 3xTg (n=6) and 3xTg;GT (n=6) mice. **i** Quantification of NCSTN immunofluorescence in hippocampal CA1 and the cortex of 9-month-old 3xTg (n=4) and 3xTg;cKO (n=4) mice. Individual values, the mean ± S.E.M, as well as P-values generated by two-tailed unpaired t-test are shown. Scale bars, 25 μm. Source data are provided as a Source Data file.

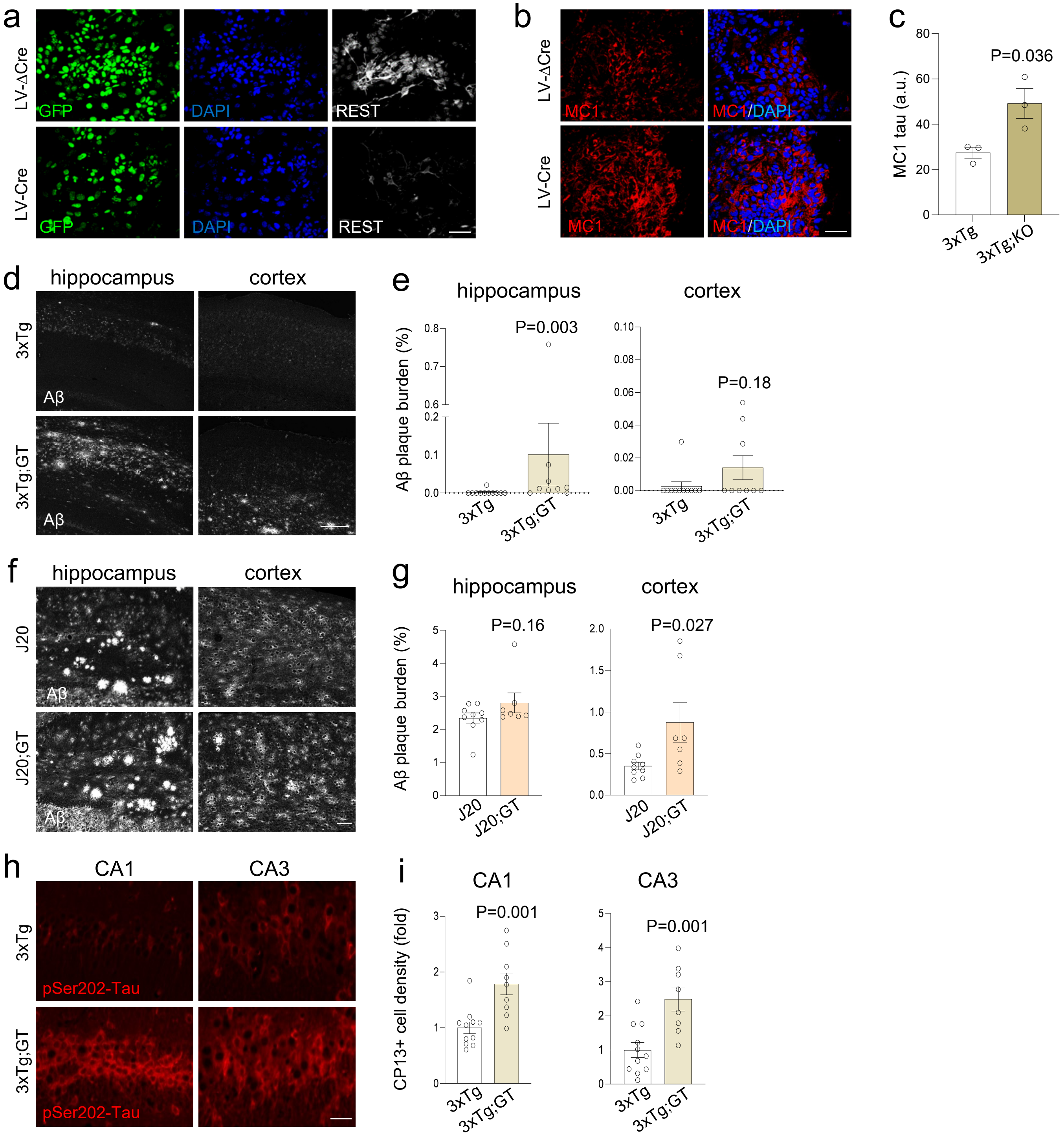

Supplementary Figure 7. REST inhibits Aβ deposition and accumulation of pTau in AD mouse models. **a** Primary cortical neuronal cultures derived from 3xTg;REST$^{lx/lx}$ mice were infected at DIV4 with recombinant lentiviruses encoding Cre recombinase or the catalytically-inactive mutant ΔCre. After one week, REST labeling shows loss of REST expression in 3xTg;REST$^{lx/lx}$ neurons infected with LV-Cre, but not LV-ΔCre. **b** REST inactivation increases the accumulation of misfolded tau (red, antibody MC1). Shown are DIV-11 primary neuronal cultures. Nuclei were labeled with DAPI (blue). **c** Quantification of cellular MC1 tau levels. The data is from n=3 independent experiments; in each experiment, the cortices of 3 embryos were pooled before the cells were dissociated and plated. **d**,**e** Loss of a single REST allele augments Aβ deposition in 3xTg mice. **d** Immunolabeling of Aβ (white) in 29-month-old hemizygous 3xTg mice shows that most animals hemizygous for 3xTg do not exhibit Aβ deposition in the hippocampus nor cortex, in agreement with previous observations14. In contrast, most 3xTg;GT mice exhibit Aβ plaques. **e** Quantification of Aβ plaque burden in the hippocampus and

cortex of 27-29 month-old 3xTg (n=11) and 3xTg;GT (n=9) mice. **f**,**g** Loss of a single REST allele augments Aβ deposition in J20 mice. **f** Immunolabeling of Aβ (white) in 29-month-old J20 and J20;GT. **g** Quantification of Aβ plaque burden in the hippocampus and cortex of 27-29-month-old J20 (n=9) and J20;GT (n=7) mice. **h**,**i** Loss of a single REST allele augments phospho-tau accumulation in 3xTg mice. **h** Immunolabeling for pSer202-tau (red, antibody CP13) in the hippocampal subfields CA1 and CA3 in 29-month-old 3xTg and 3xTg;GT mice. **i** Quantification of pSer202-tau(CP13)-positive neuronal density in 27-29-month-old 3xTg (n=11) and 3xTg;GT (n=9) mice. Individual values and the mean ± S.E.M are shown. P-values are generated by two-tailed Mann Whitney U test (**e**) or two-tailed unpaired t-test (**c**,**g**,**i**). Scale bars, 25 μm (**a**,**b**,**e**,**f**,**h**) or 125 μm (**d**). Source data are provided as a Source Data file.

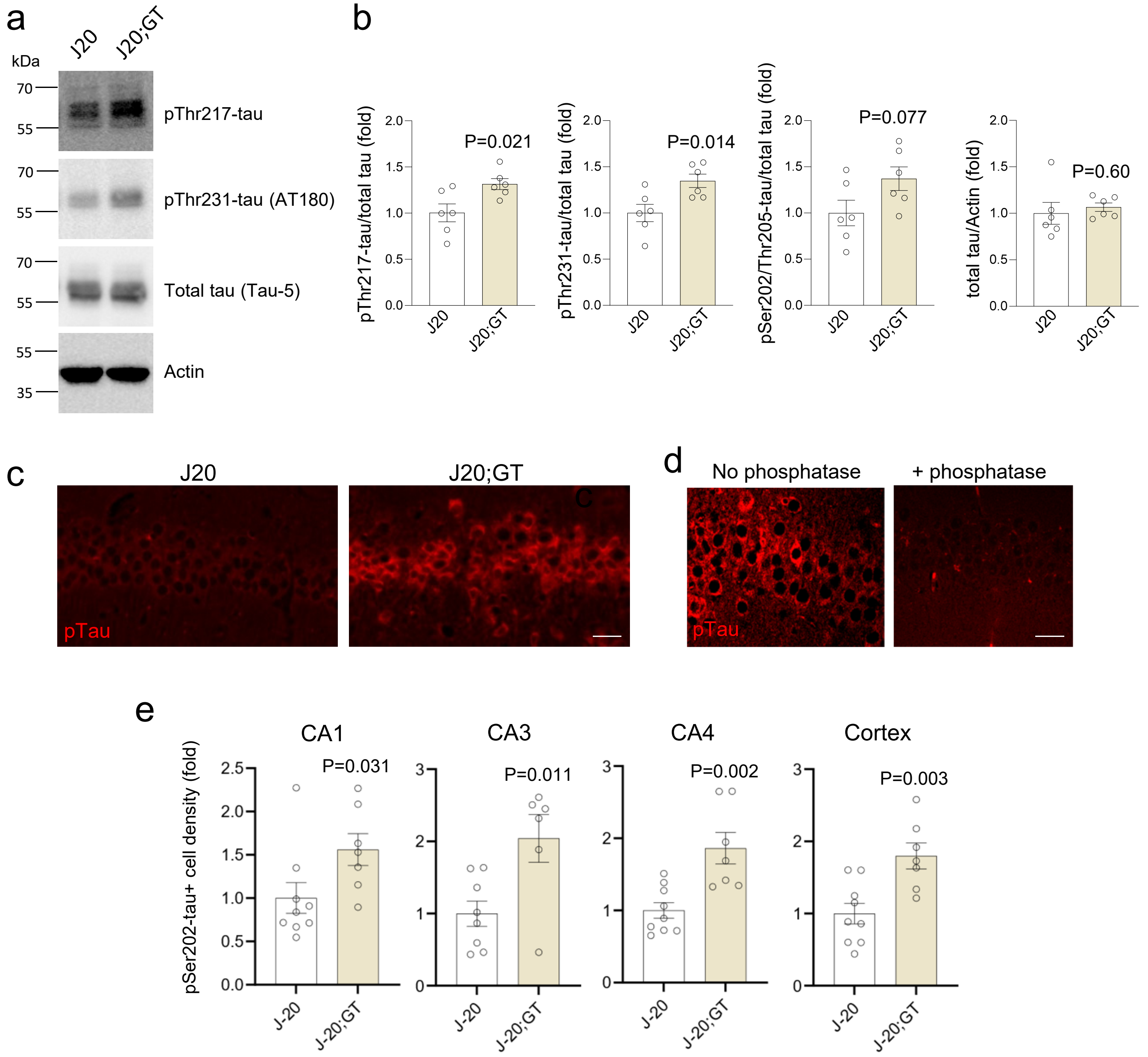

Supplementary Figure 8. Loss of REST leads to the accumulation of pathogenic tau in J-20 mice.
a,b Partial REST inactivation leads to the accumulation of multiple phospho-tau epitopes associated with AD. **a** Western blot analysis of phospho-tau isoforms pThr217-tau, pThr231-tau (antibody AT180) and pSer202/Thr205-tau (antibody AT8), total tau (antibody tau-5) and actin in 29-month-old J20 and J20;GT mice. **b** Quantification of pThr217-tau, pThr231-tau and pSer202/Thr205-tau normalized to total tau, and total tau normalized to actin in 27-29-month-old J20 (n=6) and J20;GT (n=6) mice. **c** Immunolabeling for pSer202-tau (red, antibody CP13, pTau) shows increased accumulation of phospho-tau in hippocampal CA1 neurons in REST-deficient 29-month-old J20;GT mice compared with J20 mice. **d** Specificity of phospho-tau immunolabeling. Immunoreactivity for pSer202-tau is markedly reduced following preincubation of J20;GT tissue with lambda protein phosphatase. Shown are two consecutive sections from the same J20;GT brain sample treated with either lambda protein phosphatase or buffer prior to immunolabeling with antibody CP13. **e** Quantification of pSer202-tau-positive neurons in the hippocampus (CA1, CA3 and CA4 sectors) and cortex shows significantly elevated accumulation of phospho-tau in 27-29 month-old J20;GT (n=7,6,7,7) relative to 27-29 month-old J20 (n=9,8,9,9) mice. Shown are individual values, the mean ± S.E.M, as well as P-values generated by two-tailed unpaired t-test. Scale bars, 25 µm. Source data are provided as a Source Data file.

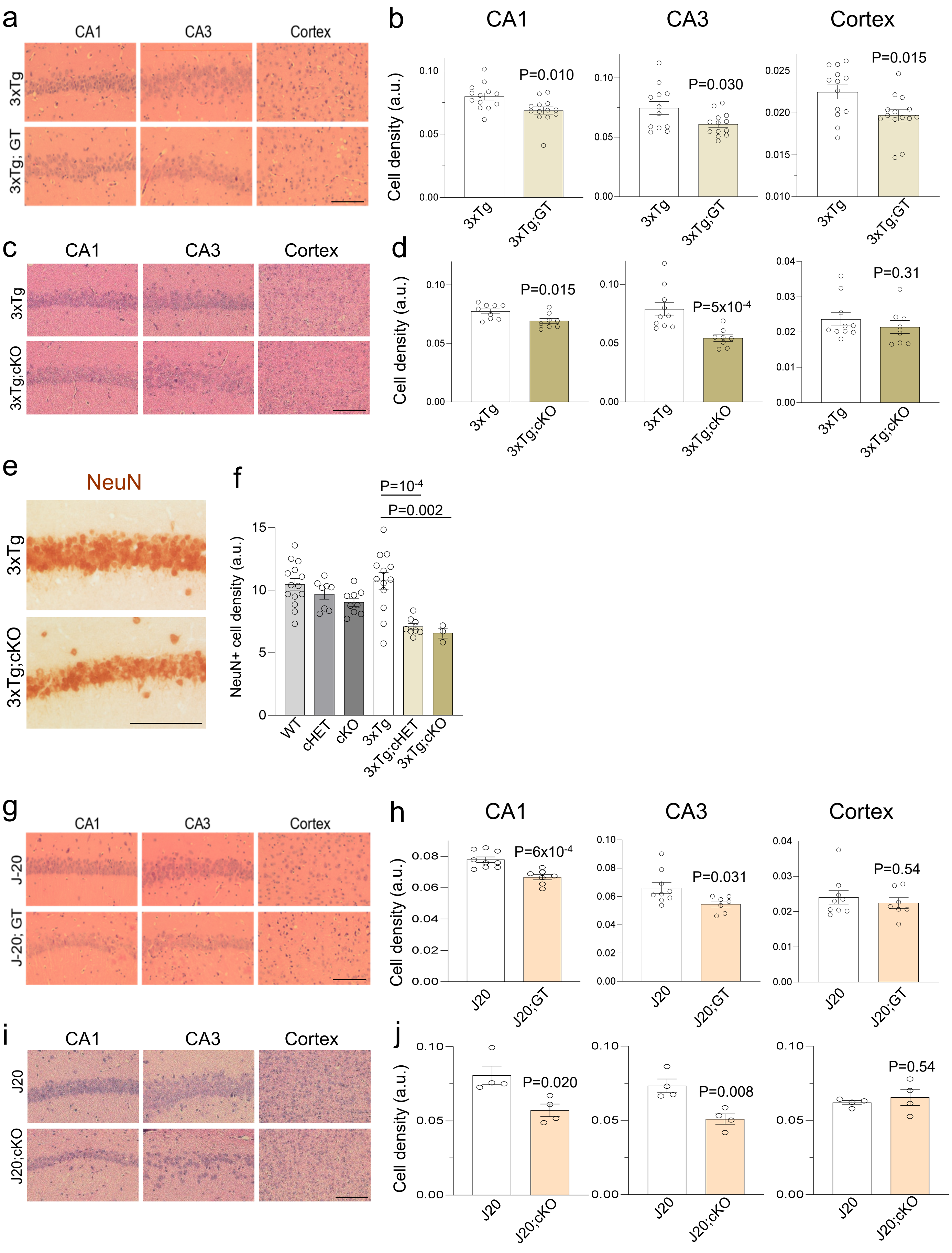
a
CA1
CA3
Cortex
3xTg
3xTg; GT
b
CA1
CA3
Cortex
Cell density (a.u.)
P=0.010
P=0.030
P=0.015
3xTg
3xTg;GT
c
CA1
CA3
Cortex
3xTg
3xTg;cKO
d
Cell density (a.u.)
P=0.015
P=5x10-4
P=0.31
3xTg
3xTg;cKO
e
NeuN
3xTg
3xTg;cKO
f
P=10-4
P=0.002
NeuN+ cell density (a.u.)
WT
cHET
cKO
3xTg
3xTg;cHET
3xTg;cKO
g
CA1
CA3
Cortex
J-20
J-20; GT
h
CA1
CA3
Cortex
Cell density (a.u.)
P=6x10-4
P=0.031
P=0.54
J20
J20;GT
i
CA1
CA3
Cortex
J20
J20;cKO
j
Cell density (a.u.)
P=0.020
P=0.008
P=0.54
J20
J20;cKO

Supplementary Figure 9. REST is neuroprotective. a,b Loss of a single REST allele leads to neuronal loss in 3xTg mice. **a** H&E labeling of hippocampal CA1 and CA3 subfields and the cortex in 29-month-old 3xTg and 3xTg;GT mice. **b** Quantification of cell density in 27-29-month-old 3xTg (n=13,12,13) and 3xTg;GT (n=13,13,14) mice. **c**,**d** Conditional deletion of REST in excitatory neurons leads to neuronal loss in 3xTg mice. **c** H&E labeling in 18-month-old 3xTg and 3xTg;cKO mice. **d** Quantification of cell density in 17-18 month-old 3xTg (n=9,10,10) and 3xTg;cKO (n=8) mice. **e**,**f** NeuN labeling confirms increased neuronal loss upon conditional deletion of one or both REST alleles. **e** NeuN labeling of hippocampal CA1 neurons in 18 month-old 3xTg and 3xTg;cKO mice. **f** Quantification of NeuN-positive neuron density in the hippocampal CA1 subfield of WT (n=14), cHET (n=8), cKO (n=9), 3xTg (n=13), 3xTg;cHET (n=8) and 3xTg;cKO (n=3) mice. Two-way ANOVA shows a significant interaction between REST genotype (+/+, cHET, cKO) and AD pathology (WT, 3xTg): $F(2, 49) = 4.87$, $P=0.011$. Shown are the adjusted P-values by two-way ANOVA with Tukey's post-hoc test. Pre-planned comparisons between 2 groups were also conducted, using two-tailed unpaired t-tests, and showed no significant difference between neuron density in WT and cHET mice ($P=0.27$); by contrast, neuronal density was significantly decreased in J20 mice relative to WT mice ($P=0.035$). **g**,**h** Loss of a single REST allele leads to neuronal loss in J20 mice. **g** H&E labeling of hippocampal CA1 and CA3 subfields and the cortex in 29-month-old J20 and J20;GT mice. **h** Quantification of cell density in 27-29 month-old J20 (n=9) and J20;GT (n=7) mice. **i**,**j** Conditional deletion of REST in excitatory neurons leads to neuronal loss in J20 mice. **i** H&E labeling of hippocampal CA1 and CA3 subfields and the cortex in 14-month-old J20 and J20;cKO mice. **j** Quantification of cell density in 14-month-old J20 (n=4) and J20;cKO (n=4) mice. Individual values and the mean ± S.E.M are shown. P-values are shown for two-tailed unpaired t-test (**b**,**g**,**h**), two-tailed Mann Whitney U test (**d**) and two-way ANOVA with Tukey's post-hoc test (**f**). Scale bars, 125 μm. Source data are provided as a Source Data file.

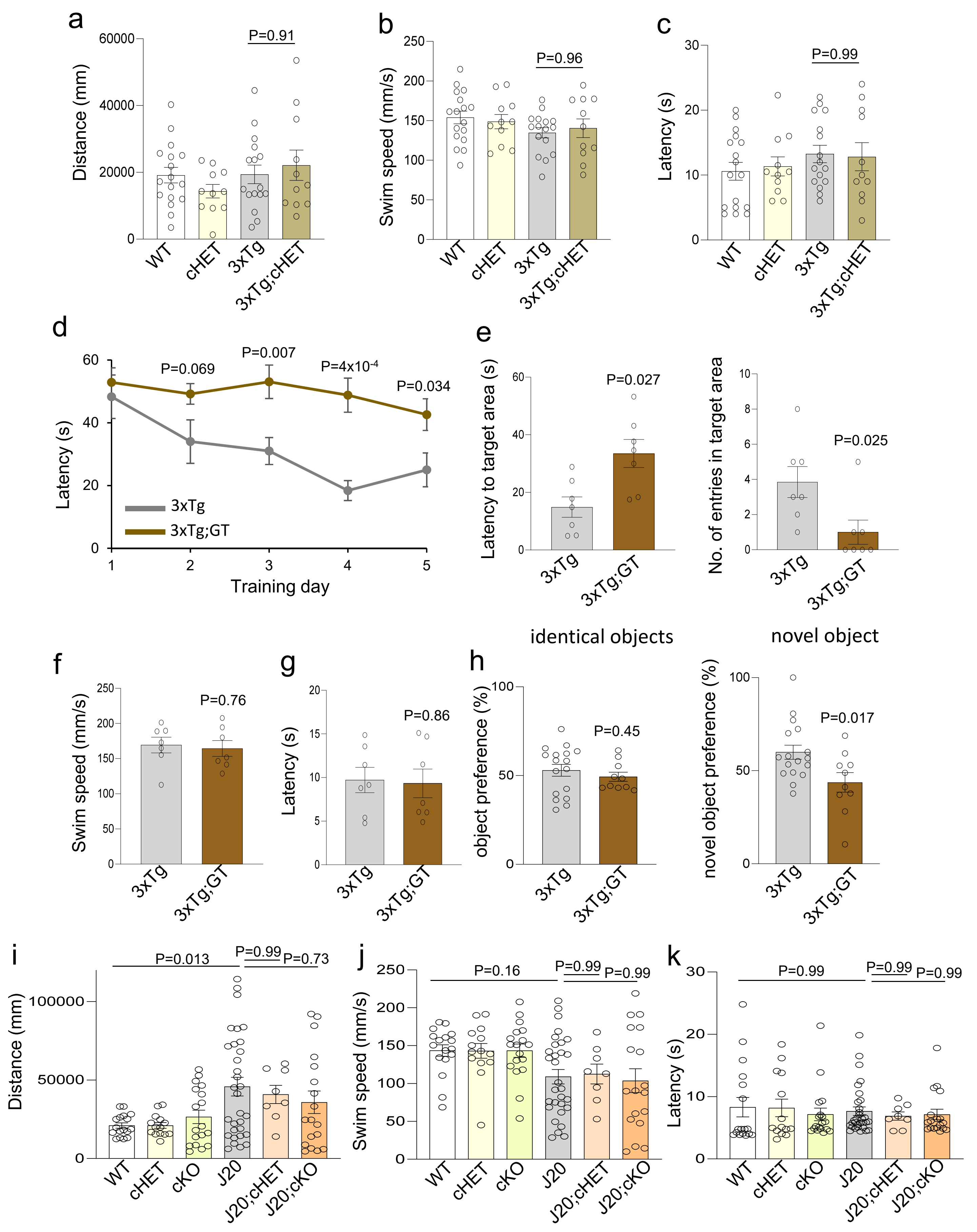
a
Distance (mm)
P=0.91
WT
cHET
3xTg
3xTg;cHET
b
Swim speed (mm/s)
P=0.96
c
Latency (s)
P=0.99
d
Latency (s)
P=0.069
P=0.007
P=4x10-4
P=0.034
3xTg
3xTg;GT
Training day
e
Latency to target area (s)
P=0.027
No. of entries in target area
P=0.025
f
Swim speed (mm/s)
P=0.76
g
Latency (s)
P=0.86
h
identical objects
object preference (%)
P=0.45
novel object
novel object preference (%)
P=0.017
i
Distance (mm)
P=0.013
P=0.99
P=0.73
WT
cHET
cKO
J20
J20;cHET
J20;cKO
j
Swim speed (mm/s)
P=0.16
P=0.99
P=0.99
k
Latency (s)
P=0.99
P=0.99
P=0.99

Supplementary Figure 10. Loss of REST accelerates cognitive decline in 3xTg and J-20 mice.
a-c Conditional inactivation of REST in 3xTg mice does not affect locomotor activity in the open field test (**a**), swim speed in the Morris water maze (**b**), or the ability of mice to locate a visible platform elevated above water level (**c**). 17-18-month-old WT (n=17), REST cHET (n=11), 3xTg (n=16), and 3xTg;cHET (n=11) mice. P-values by two-way ANOVA with Tukey's post-hoc test are shown. **d,e** 23-25 month-old 3xTg;GT mice (n=7) with partial REST deletion exhibit impaired learning (**d**) and memory retrieval (**e**) in the Morris water maze relative to 3xTg mice (n=7). **f,g** 23-25-month-old 3xTg;GT (n=7) and 3xTg mice (n=7) show similar swim speed (**f**), and ability to locate a visible platform in the Morris water maze (**g**). **h** Reduced novel object recognition in 21-23-month-old 3xTg;GT (n=10) relative to 3xTg (n=17) mice. Shown is preference for identical objects (left), and preference for a novel object (right). **i-k** Conditional inactivation of REST in 12-14-month-old J20 mice does not affect locomotor activity in the open field test (**i**), swim speed in the Morris water maze (**j**), and latency to reach a visible platform in the Morris water maze (**k**). Data was analyzed by two-way ANOVA with Tukey's post-hoc test: no statistically significant differences. We then conducted a one-way ANOVA analysis, and the adjusted P-values generated by Tukey's post-hoc test are shown. **i-k** 12-14-month-old WT (n=17), REST cHET (n=14), REST cKO (n=18), J20 (n=31), J20;cHET (n=8) and J20;cKO (n=18). Individual values, as well as the mean ± S.E.M. are shown. P-values for two-tailed unpaired t-tests (**d**-**h**) are also shown. Source data are provided as a Source Data file.

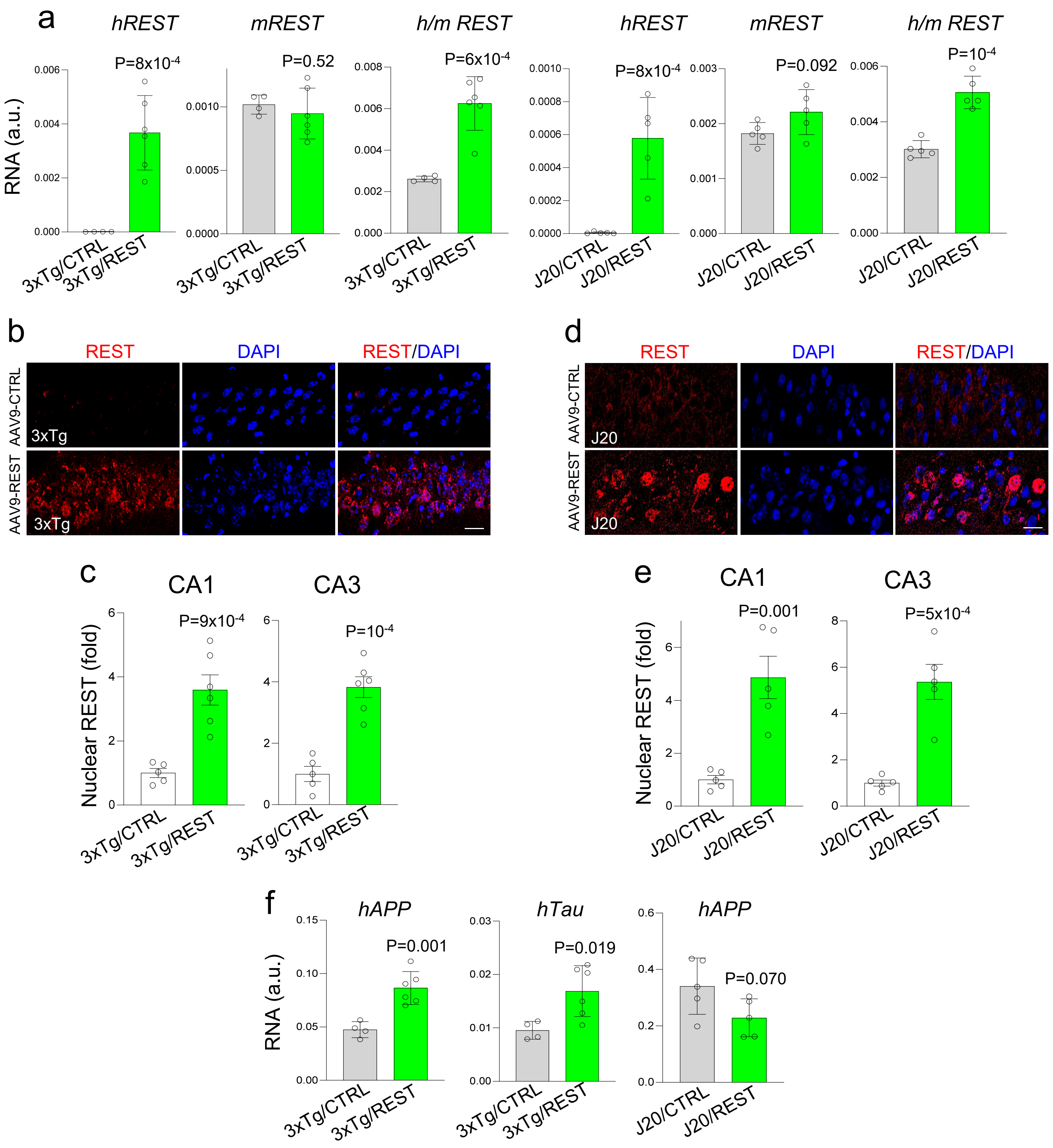

Supplementary Figure 11. Overexpression of human REST in the hippocampus of AD mice.
a qRT-PCR analysis of human REST (hREST) and mouse REST (mREST) RNA levels in the hippocampus of 3xTg or J20 mice that received intracranial injections of AAV9-REST or AAV9-CTRL at the ages of 11 and 14 months, respectively, and were sacrificed 10 weeks later. Primers were also used to amplify a REST mRNA region conserved between mouse and human REST (h/m REST). **b** Immunofluorescent labeling of REST (red) and DNA (blue) in the hippocampus of 3xTg mice that had received an intracranial delivery of AAV9-REST or AAV9-CTRL at the age of 11 months and were sacrificed 10 weeks later. Note the increased in nuclear REST expression in the 3xTg/AAV9-REST hippocampus. **c** Quantification of nuclear REST levels in the CA1 and CA3 regions of the hippocampus in n=5 3xTg/AAV9-CTRL and n=6 3xTg/AAV9-REST mice, expressed as mean

fluorescence intensity; a.u.-arbitrary units. **d** Immunofluorescent labeling of REST (red) and DNA (blue) in the hippocampus of J20 mice that had received an intracranial delivery of AAV9-REST or AAV9-CTRL at the age of 14 months and were sacrificed 10 weeks later. An antibody that detects human REST (rabbit polyclonal, Bethyl, Catalog No. IHC-00141) was used. Note the increased in nuclear REST expression in the J20/AAV9-REST hippocampus. **e** Quantification of nuclear REST levels in the CA1 and CA3 regions of the hippocampus in n=5 J20/AAV9-CTRL and n=5 J20/AAV9-REST mice, expressed as mean fluorescence intensity; a.u.-arbitrary units. **f** qRT-PCR analysis of human APP and MAPT(TAU) transgene expression in the hippocampus of 3xTg or J20 mice that received intracranial injections of AAV9-REST or AAV9-CTRL at the ages of 11 and 14 months, respectively, and were sacrificed 10 weeks later. Individual values, the mean ± S.E.M., and P-values generated by two-tailed unpaired t-tests are shown. Scale bars, 25 μm. Source data are provided as a Source Data file.

Uncropped blots for data presented in Supplementary Figures

Supplementary Figure 5c

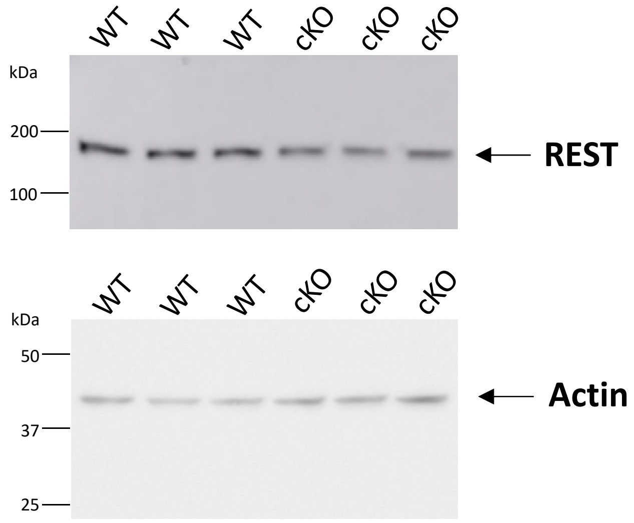

Supplementary Figure 5h

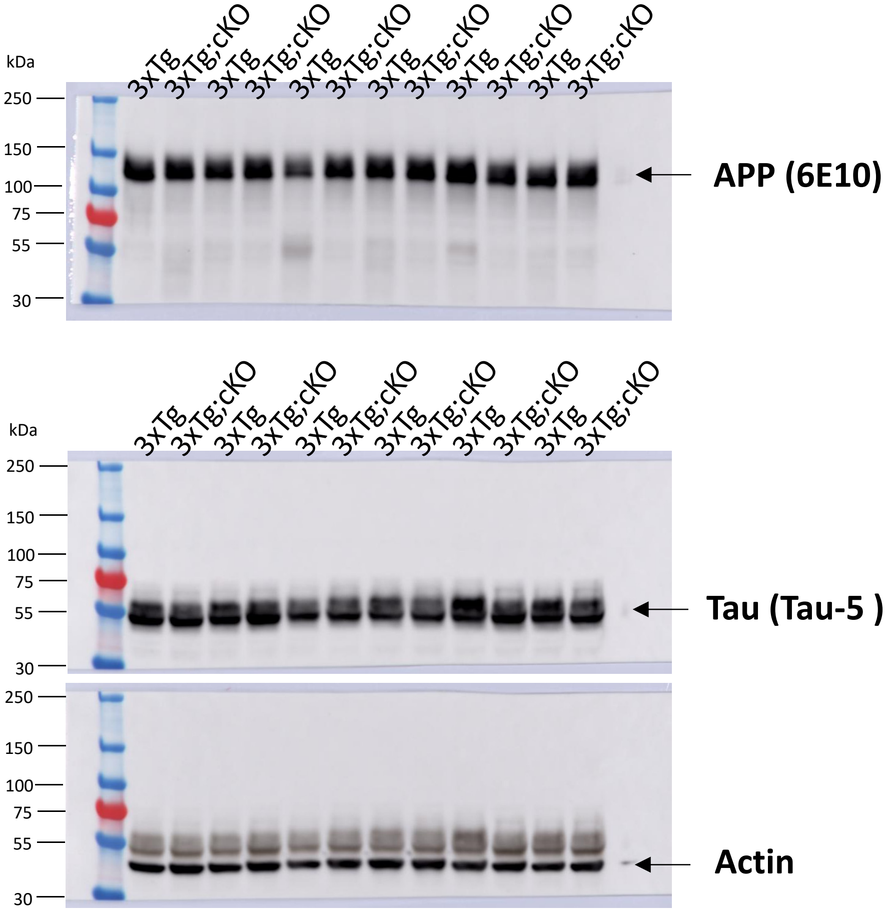

Supplementary Figure 5k

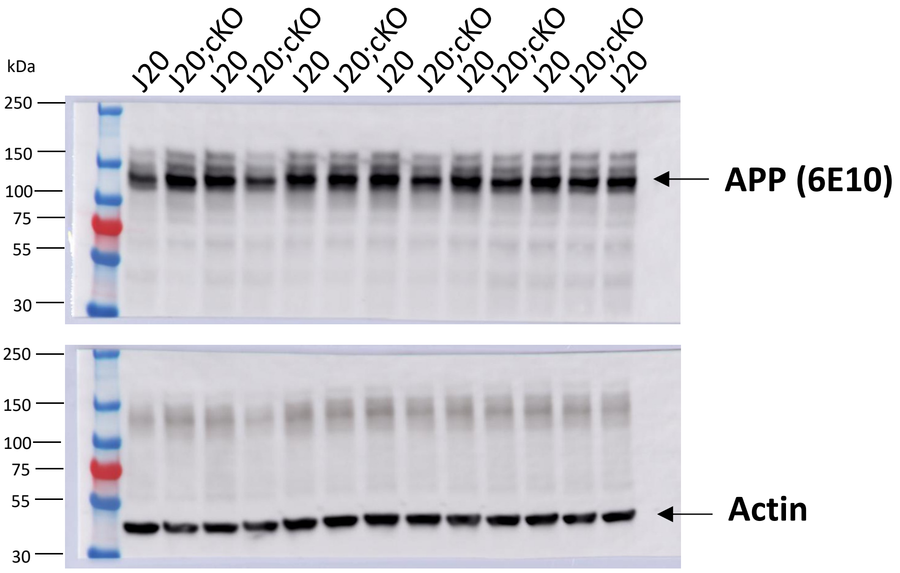

Supplementary Figure 8a

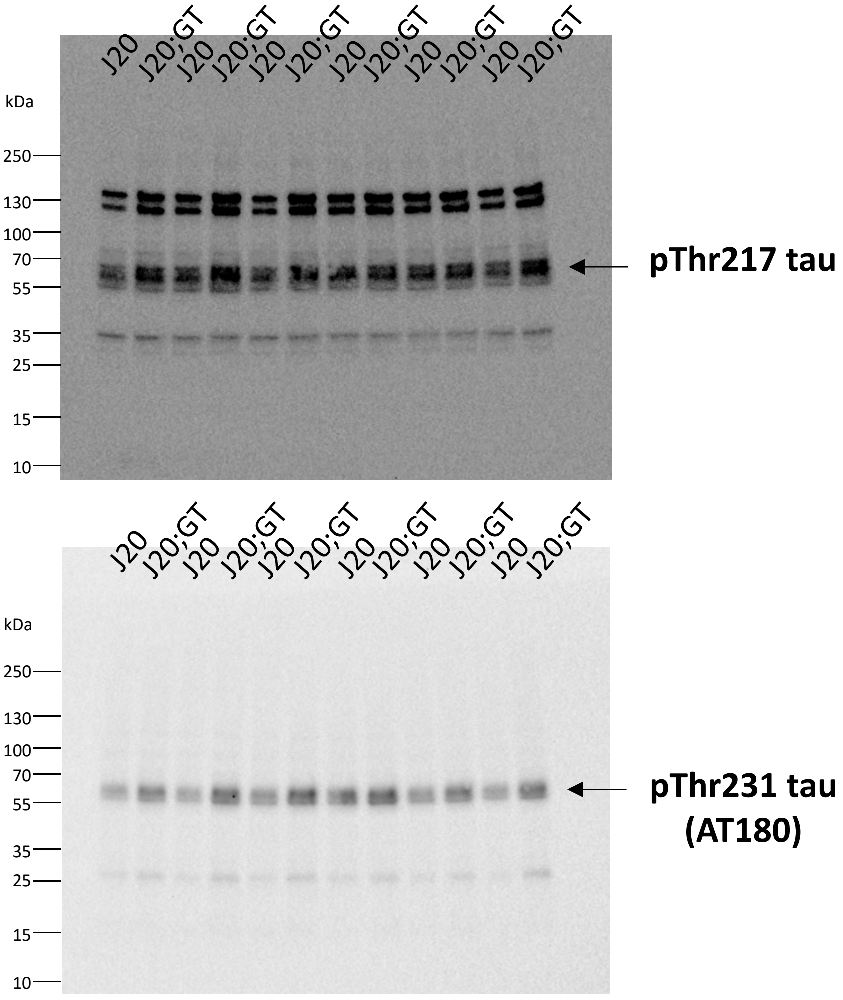

Supplementary Figure 8a

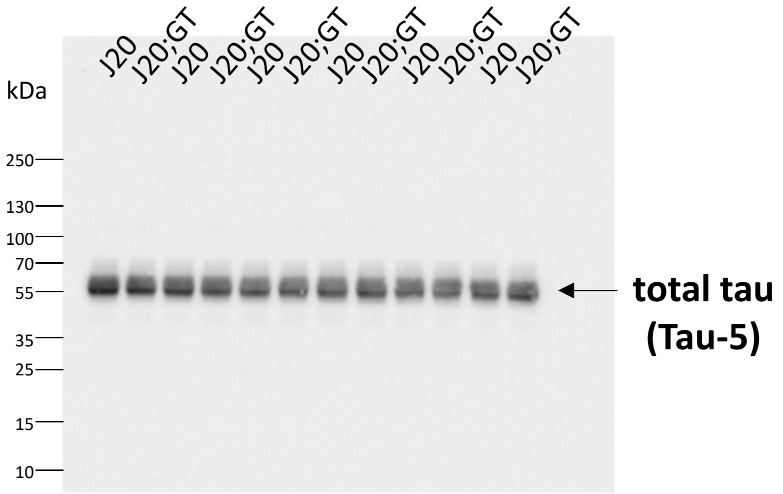

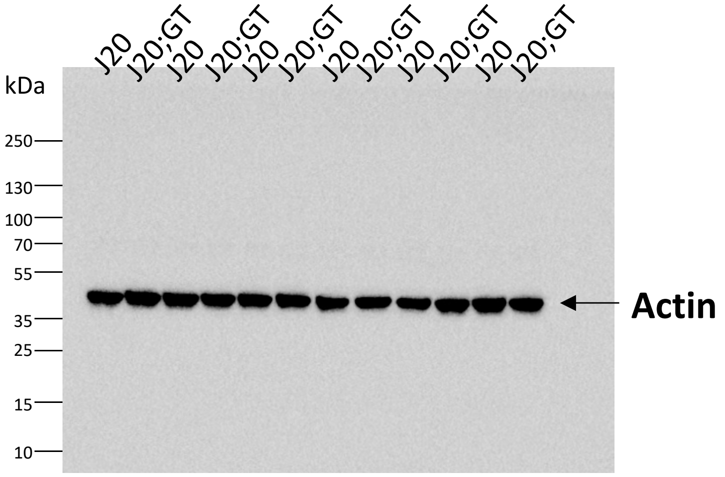

Supplement: Supplementary file 1 — Supplementary Information [file 41467_2023_42704_MOESM1_ESM.pdf]
