# Supplementary material for: A neurodegeneration checkpoint mediated by REST protects against the onset of Alzheimer’s disease
Source: Nat Commun. 2023 Nov 2;14:7030. doi: 10.1038/s41467-023-42704-6 (PMC10622455; doi:10.1038/s41467-023-42704-6)
Supplement: Supplementary file 3 — Description of Additional Supplementary Files [file 41467_2023_42704_MOESM3_ESM.docx]

**Description of Additional Supplementary Files**

**Supplementary Data 1.** Summary of statistical tests.

**Supplementary Data 2.** Summary of the ChIP-seq experiment.

**Supplementary Data 3.** List of ChIP-seq peaks in WT (A) and 3xTg (B) mouse cortex.

**Supplementary Data 4.** List of peaks common to WT and 3xTg mice (A) and gene ontology enrichments (B).

**Supplementary Data 5.** Differential binding analysis: List of peaks (A) and genes (B) exhibiting significantly increased REST binding in 3xTg cortex, and gene ontology analysis (C).

**Supplementary Data 6.** PCR primer sequences.

**Supplementary Data 7.** List of reagents.

**Supplementary Data 8.** REST predicts global cognitive function during aging. Multivariate regression analyses of global cognitive function (dependent variable) as a function of (a) nuclear REST levels in cortical pyramidal neurons; (b) age and REST cortical levels; (c) age, AD pathology, and REST levels in cortical neurons; (d) age, neuropathology, and REST cortical levels; (e) age, gender, apoE4 genotype, education, AD pathology, and REST cortical levels; or (f) age, gender, apoE4 genotype, education, neuropathology, and REST cortical levels. Age, gender, education (in years), the presence of an apoE4 allele, measures of neuropathology were obtained for n=145 individuals from ROSMAP, aged 70-102, comprising n=82 NCI cases and n=63 AD cases, for which nuclear REST nuclear levels in cortical pyramidal neurons were assessed by immunofluorescence.
