# Supplementary material for: A neurodegeneration checkpoint mediated by REST protects against the onset of Alzheimer’s disease
Source: Nat Commun. 2023 Nov 2;14:7030. doi: 10.1038/s41467-023-42704-6 (PMC10622455; doi:10.1038/s41467-023-42704-6)
Supplement: Supplementary file 12 — Reporting Summary [file 41467_2023_42704_MOESM12_ESM.pdf]

nature portfolio

Corresponding author(s): Bruce A. Yankner

Last updated by author(s): Oct 9, 2023

Reporting Summary

Nature Portfolio wishes to improve the reproducibility of the work that we publish. This form provides structure for consistency and transparency in reporting. For further information on Nature Portfolio policies, see our Editorial Policies and the Editorial Policy Checklist.

Statistics

For all statistical analyses, confirm that the following items are present in the figure legend, table legend, main text, or Methods section.

n/a	Confirmed	
☐	☒	The exact sample size (*n*) for each experimental group/condition, given as a discrete number and unit of measurement
☐	☒	A statement on whether measurements were taken from distinct samples or whether the same sample was measured repeatedly
☐	☒	The statistical test(s) used AND whether they are one- or two-sided<br>*Only common tests should be described solely by name; describe more complex techniques in the Methods section.*
☐	☒	A description of all covariates tested
☐	☒	A description of any assumptions or corrections, such as tests of normality and adjustment for multiple comparisons
☐	☒	A full description of the statistical parameters including central tendency (e.g. means) or other basic estimates (e.g. regression coefficient) AND variation (e.g. standard deviation) or associated estimates of uncertainty (e.g. confidence intervals)
☐	☒	For null hypothesis testing, the test statistic (e.g. *F*, *t*, *r*) with confidence intervals, effect sizes, degrees of freedom and *P* value noted<br>*Give P values as exact values whenever suitable.*
☒	☐	For Bayesian analysis, information on the choice of priors and Markov chain Monte Carlo settings
☒	☐	For hierarchical and complex designs, identification of the appropriate level for tests and full reporting of outcomes
☐	☒	Estimates of effect sizes (e.g. Cohen's *d*, Pearson's *r*), indicating how they were calculated

Our web collection on statistics for biologists contains articles on many of the points above.

Software and code

Policy information about availability of computer code

Data collection	For the analysis of immunofluorescence pictures, we used an Olympus FluoViewTM LV1000 confocal microscope and 20x or 40x objectives, coupled to a camera. For H&E- or DAB-stained sections, we acquired pictures using a brightfield microscope coupled to a camera. Western blotting chemiluminescence data was collected using Bio Rad's ChemiDoc MP. Behavioral data was collected by monitoring the mice in real time, using the TopScanLite software from CleverSys Inc., coupled to a camera. Behavioral data was collected automatically for each animal. Real time PCR data was collected using Bio Rad's CFX Connect Real-Time PCR detection system. For ChIP-seq, DNA libraries were sequenced on Illumina's NextSeq 500.

Data analysis	Multiple linear regression models, as well as all other statistical analyses were performed using the GraphPad software (version 9.3.1). For analysis of Aβ plaque burden, as well as CP13+ or MC1+ tau cell density, pictures of Aβ immunoreactivity, MC1-tau, or CP13+ tau immunoreactivity were processed using a macro developed for use with Fiji/ImageJ v2.9.0 (see Methods for macro settings). Western blot pictures were processed in ImageJ, and band intensities were measured using the Analyze/Gels menu. Behavioral data was analyzed using Microsoft Excel and Graph Pad software (version 9.3.1). ChIP-seq reads were aligned to the mouse genome (mm10) using the STAR algorithm46 (v2.7.0a). Peak locations were determined using the MACS algorithm (v2.1.0) and normalized fold enrichment tracks were generated by using the callpeak function with --SPMR, then passing the bedgraph outputs into the bdgcmp function with the setting -m FE (fold enrichment). Bedgraph files were converted into bigWig and visualized using Integrated Genome Viewer (IGV, v2.4). Peaks were filtered for fold enrichment more than 5 and q-value less than 0.01; furthermore, peaks that were on the ENCODE blacklist of known false ChIP-seq peaks were removed. Motif enrichment analysis was performed using HOMER. Bedtools Intersect was used to overlap peaks from WT and 3xTg samples to determine common regions in each group. Differential binding sites was identified using DiffBind using default settings. Peaks were annotated using ChIPseeker, computeMatrix (v3.3.2) was applied to build a matrix with a flanking region of ± 1 kb around the center of enriched REST peaks and plotHeatmap (v3.3.2) was used to generate heatmaps. We employed DAVID for functional enrichment analysis and enrichment of gene ontology (GO) terms was calculated using Fisher's exact test with Bonferroni correction.

For manuscripts utilizing custom algorithms or software that are central to the research but not yet described in published literature, software must be made available to editors and reviewers. We strongly encourage code deposition in a community repository (e.g. GitHub). See the Nature Portfolio guidelines for submitting code & software for further information.

Data

Policy information about availability of data

All manuscripts must include a data availability statement. This statement should provide the following information, where applicable:

- Accession codes, unique identifiers, or web links for publicly available datasets
- A description of any restrictions on data availability
- For clinical datasets or third party data, please ensure that the statement adheres to our policy

Clinico-pathological data on post-mortem human samples from ROSMPAP can be requested at https://www.radc.rush.edu. ChIP-seq data has been deposited at Gene Expression Omnibus (GEO): ncbi.nlm.nih.gov/geo/query/acc.cgi?acc=GSE195446 (accession code GSE195446). Source data are provided with this paper.

Human research participants

Policy information about studies involving human research participants and Sex and Gender in Research.

Reporting on sex and gender	Post-mortem brain tissue from both self-reported males and females was included in the study. Information about the frequency of males and females in the samples that were analyzed can be found in the Methods Section under "Brain sample procurement and description".
Population characteristics	Postmortem human brain material was procured in accordance with institutional guidelines and was approved by the Harvard Medical School Institutional Review Board. Tissue samples were procured from the Rush Alzheimer's Disease Center (RADC). Tissue samples (both paraffin-embedded and frozen) from the RADC were derived from participants in the Religious Orders Study (ROS) or Rush Memory and Aging Project (MAP) (together referred to as ROSMAP) at the RADC; these are longitudinal, clinical–pathologic studies of aging, cognitive decline and AD. Study participants agreed to comprehensive annual clinical and neuropsychological evaluation and to brain donation at death. Informed consent, an Anatomic Gift Act, and a repository consent were obtained and the study was approved by an Institutional Review Board (IRB) of Rush University Medical Centre. ROSMAP data can be requested at https://www.radc.rush.edu.<br><br>To assess cognitive function, 21 cognitive function tests were used, 11 of which directly informed on clinical diagnoses of Alzheimer's dementia, mild cognitive impairment (MCI) and no cognitive impairment (NCI) as previously described38,39. A global measure of cognition was computed from 19 independent test scores (7 assessments of episodic memory, 3 measures of semantic memory, 3 measures of working memory, 2 measures of perceptual orientation, and 4 measures of perceptual speed; see https://www.radc.rush.edu/docs/var/detail.htm?category=Cognition&subcategory=Global+cognition&variable=cogn_global)). APOE genotype was determined as previously reported. The follow-up rate exceeds 95% and the autopsy rate exceeds 90%.<br><br>All individuals who underwent autopsy were subject to a uniform structured neuropathologic evaluation of AD, including assessment of global pathology, a quantitative summary of AD pathology derived from standardized counts of neuriticand diffuse plaques, and neurofibrillary tangles, determined by microscopic examination of silver-stained slides from 5 regions, Neuritic plaque pathology (CERAD score), the density and distribution of neurofibrillary tangles (Braak stage), and a composite measure of neurofibrillary tangles and neuritic plaques (NIA/Reagan score) (https://www.radc.rush.edu/docs/var/detail.htm;jsessionid=329C70639AA50EAE971DBEC631D06872?category=Pathology&subcategory=Alzheimer%27s+disease&variable=gpath). Measures of neuropathology at AD pathology also included β-amyloid load and PHFtau tangle density from immunostaining of 8 brain regions. In addition, we examined Lewy bodies, and neocortical, macroinfarcts and microinfarcts as previously reported.<br><br>Using a sample of n=518 NCI cases and n=501 Alzheimer's dementia cases from the ROSMAP cohort, we stratified the cases based on clinical diagnosis (NCI or AD) and the stage of AD pathology, as reflected by the global AD pathological burden (gpath): no AD pathology (gpath values below the 15th percentile: 0 ≤ gpath≤ 0.09), early AD pathology (gpath values between the 15th and the 40th percentile: 0.09< gpath≤0.49), mid AD pathology (gpath values between the 40th and the 75th percentile: 0.49< gpath≤1.2) and late AD pathology (gpath values above the 75th percentile: gpath >1.2). The differences between the 4 stages of pathology were statistically significant for the measure of global pathology (gpath), as

	well as CERAD, Braak and NIA/Reagan scores (Supplementary Fig. 1a). The distribution of each level of AD pathology among NCI and AD cases is shown in Supplementary Fig. 1b.
Recruitment	No donors were recruited, the tissue has been obtained from participants in the Religious Order Study.
Ethics oversight	The Religious Orders Study and Rush Memory and Aging Project were approved by an IRB of Rush University Medical Center.

Note that full information on the approval of the study protocol must also be provided in the manuscript.

Field-specific reporting

Please select the one below that is the best fit for your research. If you are not sure, read the appropriate sections before making your selection.

☒ Life sciences ☐ Behavioural & social sciences ☐ Ecological, evolutionary & environmental sciences

For a reference copy of the document with all sections, see nature.com/documents/nr-reporting-summary-flat.pdf

Life sciences study design

All studies must disclose on these points even when the disclosure is negative.

Sample size	No statistical methods were used to pre-determine sample size. Sample sizes were chosen based on prior literature that used similar approaches (see Martorell et al. Cell 177(2):256-271 (2019); Zullo et al. Nature 574, 359-364 (2019); Iaccarino et al. Nature 540(7632):230-235 (2016); Sevigny et al. Nature 537(7618):50-6 (2016); Oddo et al. Neuron 39(3):409-21 (2003)). The sample size was determined to be adequate based on the magnitude and reproducibility of observed differences between the different groups.
Data exclusions	Statistical outliers were identified using the GraphPad software using the ROUT method (Q=1 %, equivalent to a False Discovery Rate FDR<1%) to identify any outliers from nonlinear regression. No data were excluded from the analyses.
Replication	The pathological findings (such as increased amyloid and tau deposition upon genetic inactivation of REST) were replicated several times, by using 2 independent AD mouse models (3xTg and J20), two independent REST loss-of-function alleles (Cre/LoxP-derived conditional allele, and REST gene trap null allele) and a combination of immunofluorescence labeling and Western blotting. We also generated animals with a partial or complete REST deletion (heterozygous or homozygous conditional REST KO) and the findings in REST heterozygous knockouts (Cre/LoxP-derived allele, or genetrap allele) were the same as those seen with conditional REST homozygous knockouts. For some experiments, we generated and analyzed mice at two time points during the aging process (e.g. 3xTg and 3xTg;cKO at 9 and 17-18 months).<br><br>The behavioral findings in this paper (accelerated cognitive decline upon deletion of REST in AD mouse models) were replicated independently in 3 different mouse cohorts: 3xTg, 3xTg;cHET (cohort 1, Figure 8b,c), 3xTg and 3xTg;GT (cohort 2, Extended Data Fig 10), and J20, J20;cHET, J20;cKO (cohort 3, Figure 8d,e). The cognitive impairment resulting from loss of REST was shown using both the Morris water maze and the novel object recognition behavioral paradigms (Figure 8 and Supplementary Fig. 10).<br><br>The findings that loss of REST accelerates neurodegeneration in AD mouse models were similarly replicated in 4 cohorts: 3xTg vs. 3xTg;GT (cohort 1, Suppl Fig 9a,b), 3xTg vs. 3xTg;cKO (cohort 2, Suppl Fig 9c,d), J-0 vs J20;GT (cohort 3, Suppl Fig 9g,h, and J20 vs. J20;cKO (cohort 4, Suppl Fig 9i,j). Moreover, we used both H&E labeling and NeuN labeling to show that loss of REST leads to neurodegeneration (Suppl Fig. 9) and we provided further proof of neurodegeneration using TUNEL to label apoptotic cells (Figure 8a).<br><br>The unbiased ChIP-seq results were replicated by ChIP-qPCR for selected target genes, using 2 independent animal cohorts (aged 5 and 11 months; Figure 4e).<br><br>The major mechanistic findings (REST suppresses the generation of amyloid beta and the expression of the major tau kinases CDK5 and GSK3beta, leading to decreased abnormal tau phosphorylation) were replicated as follows: (1) the suppression of gamma secretase components by REST was shown in AD mice (Suppl Fig. 6), human neural cells, as well as MEF cells (Figure 6). (2) the regulation of amyloid beta production by REST was shown via gene manipulation in AD mice carrying REST loss-of-function alleles (Figure 7, Suppl Fig. 7) as well as via AAV-mediated REST overexpression in vivo (Figure 9), and in vitro in REST KO MEF cells that do not carry any AD transgenes (Figure 6). (3) the regulation of CDK5 and GSK3beta expression by REST was shown in 2 independent mouse cohorts (3xTg vs. 3xTg;cKO and 3xTg vs. 3xTg;GT) by immunofluorescence and Western blotting in REST knockout mice (Figure 5) and also in 3xTg mice that received AAV9-REST (Figure 9). (4) the regulation of tau phosphorylation by REST was shown via gene manipulation in AD mice carrying REST loss-of-function alleles (Figure 7, Suppl Figures 7, 8) as well as via AAV-mediated REST overexpression in vivo (Figure 9), and in vitro in REST knockout 3xTg primary neurons (Suppl Fig 7).<br><br>The effects of human REST gene delivery via AAV was replicated in 2 AD mouse models (Figure 9).
Randomization	Postmortem human samples (n=82 NCI and n=63 AD cases) were randomly selected from a larger pool of ROSMAP samples, based on pre-set criteria that were equally applied to all samples: age (70-102), low postmortem intervals (typically 6-10 hours or less) and the availability of paraffin-embedded tissue. The randomly-selected postmortem samples were then allocated to the experimental groups based on the cognitive status of each case -- no cognitive impairment (cogdx=1; group 1) vs. Alzheimer's disease (cogdx=4; group 2). Within each of these 2 groups, the samples were further sub-divided into 4 groups (no pathology, early pathology, mid pathology and late pathology) using the following cutoffs for the global AD pathological burden (gpath) variable: no AD pathology ($0 \leq$ gpath≤ 0.09), early AD pathology ($0.09<$ gpath≤ 0.49), mid AD pathology ($0.49<$ gpath≤ 1.2) and late AD pathology (gpath >1.2).<br>Mice were randomly allocated to experimental groups (such as behavioral studies, histology, biochemistry).

Blinding	For the analysis of mouse samples (including histological, biochemical and behavioral samples), the investigators were blind to sample genotypes. For the analysis of postmortem human samples, the investigators were blind to sample diagnosis.

Reporting for specific materials, systems and methods

We require information from authors about some types of materials, experimental systems and methods used in many studies. Here, indicate whether each material, system or method listed is relevant to your study. If you are not sure if a list item applies to your research, read the appropriate section before selecting a response.

Materials & experimental systems

n/a	Involved in the study
☐	☒ Antibodies
☐	☒ Eukaryotic cell lines
☒	☐ Palaeontology and archaeology
☐	☒ Animals and other organisms
☒	☐ Clinical data
☒	☐ Dual use research of concern

Methods

n/a	Involved in the study
☐	☒ ChIP-seq
☒	☐ Flow cytometry
☒	☐ MRI-based neuroimaging

Antibodies

Antibodies used

The following antibodies were used to detect the REST protein: (1) a rabbit polyclonal IgG that recognizes a region between residues 1050-1097 (C-terminus) of REST (Bethyl laboratories, IHC-00141). (2) The REST C-terminal antibody used for ChIP-seq was a gift from Gail Mandel (Vollum Institute). Reference: Ballas et al. Cell 121, 645-657 (2005). This antibody has previously been used for ChIP (see McGann et al. J. Neurosci 41, 6582-6595 (2021). (3) For ChIP-qPCR we used a second anti-REST antibody, as well as non-specific IgG control (both from Millipore Sigma, Catalog No. 17-641). (4) For detection of mouse REST by IHC/IF, we employed the rabbit polyclonal antibody REST14, a generous gift from Jenny Hsieh (University of Texas at San Antonio).

To detect different tau species, we used the following antibodies: (1) a mouse monoclonal antibody that recognizes phosphorylated Ser 202 tau (CP13 clone; generous gift from Peter Davies, Albert Einstein College of Medicine, NY). (2) a mouse monoclonal antibody raised against a conformationally altered form of tau in AD (MC1 clone; from Peter Davies). (3) a mouse monoclonal antibody raised against phosphorylated Ser 396 tau (PHF1 clone; from Peter Davies). (4) a mouse monoclonal antibody that recognizes phosphorylated Ser202/Thr205 tau (clone AT8; ThermoFisher Scientific, Catalog No. MN1020). (5) a mouse monoclonal antibody that recognizes phosphorylated Thr231 tau (clone AT180; ThermoFisher Scientific, Catalog No. MN1040). (6) a rabbit polyclonal antibody that recognizes phosphorylated Thr217 tau (ThermoFisher Scientific, Catalog No. 44-744). (7) a mouse monoclonal antibody that recognizes all tau species (total tau; clone tau-5; ThermoFisher Scientific, Catalog No. AHB0042).

Additional primary antibodies were as follows: anti-human Aβ rabbit monoclonal IgG antibody (Cell Signaling, Cat. No. 8243); anti-human APP mouse monoclonal IgG antibody (clone 6E10; Covance, Catalog No. SIG-39320); anti-actin mouse monoclonal IgG antibody (clone ACTN05 (C4); ThermoFisher Scientific, Catalog No. MA5-11869); anti-NeuN mouse monoclonal IgG antibody (clone A60, Millipore, MAB377); anti-MAP2 goat polyclonal IgG antibody (PhosphoSolutions, Catalog. No. 1099-MAP2); anti-CDK5 mouse monoclonal IgG antibody (clone 4E4; Novus Bio, Catalog No. NBP2-37602); anti-GSK3β mouse monoclonal IgG antibody (clone D5C5Z; Novus Bio, catalog No. NBP1-47470S); anti-PS1 C-terminal (CTF) rabbit monoclonal IgG antibody (clone EP2000Y; Abcam, Catalog No. ab76083); anti-PS1 N-terminal (NTF) rabbit polyclonal IgG antibody (231-f; made in the Yankner lab); anti-Nicastrin mouse monoclonal IgG antibody (clone 9C3; Biolegend, Catalog No. 852301); anti-Nicastrin rabbit polyclonal IgG antibody (Sigma Millipore, Catalog No. N1660); anti-PEN2 rabbit polyclonal IgG antibody (ProScience, Catalog No.3981); anti-PEN2 rabbit monoclonal IgG antibody (clone EPR9200; Abcam, Catalog No. ab154830); anti-PEN2 rabbit polyclonal IgG (ProScience, Catalog No. 3981); anti-Transferrin receptor mouse monoclonal IgG antibody (clone H68.4; ThermoFisher Scientific, Catalog No. 13-6800); anti-BiP/GRP78 mouse monoclonal IgG (clone C38; ThermoFisher Scientific, clone C38, Catalog No. 14-9768-82); anti-β-catenin goat polyclonal IgG(R&D Systems, Catalog No. AF1329); non-specific rabbit IgG antibody (Sigma Millipore, Catalog No. 17-641); and anti-FLAG mouse monoclonal IgG1 antibody (clone M2; Sigma Millipore, Catalog No. F3165).

The secondary antibodies were as follows: Alexa Fluor 647 donkey anti-rabbit IgG (ThermoFisher Scientific, Catalog No. A31573); Alexa Fluor 488 donkey anti-rabbit IgG (ThermoFisher Scientific, Catalog No. A21206); Alexa Fluor 694 donkey anti-mouse IgG (ThermoFisher Scientific, Catalog No. A21203); Alexa Fluor 647 donkey anti-goat IgG (ThermoFisher Scientific, Catalog No. A21447).

Validation

The specificity of the anti-REST antibody (Bethyl, IHC-00141), which we used to label REST in post-mortem human brain tissue, was confirmed by us previously (Lu et al. Nature 507(7493):448-54 (2014)). Briefly, labeling of human neural cells in which REST was knocked down or overexpressed led to a significant loss, and a significant increase, in REST nuclear immunolabeling, respectively. The qualitative and quantitative pattern of REST immunolabeling for the REST Bethyl IHC-00141 antibody was very similar to those obtained with 2 independent REST antibodies by immunofluorescence (Lu et al. 2014). When the REST Bethyl IHC-00141 antibody was pre-incubated with excess of the REST immunizing (blocking) peptide (corresponding to aa 1000-1097 of REST), REST immunolabeling was largely abrogated (Lu et al. 2014 and see Supplementary Fig. 1c). No signal was detected when the REST Bethyl IHC-00141 primary antibody was omitted from the staining protocol (data not shown).

The REST C-terminal antibody used for ChIP-seq (a gift from Gail Mandel) has previously been used for ChIP (see McGann et al. J. Neurosci 41, 6582-6595 (2021). We also used this antibody to detect REST in WT and Nestin-Cre; REST(lx/lx) mice. Western blot analysis of cortices from WT and REST brain conditional KO adult mice showed robust REST labeling in WT mice, and loss of full-length REST immunoreactivity in REST conditional KO mice (data not shown).

The anti-REST antibody from Sigma Millipore (Catalog No. 17-641) has been validated for ChIP by Sigma Millipore as indicated on the antibody page: "All ChIPAb+ antibodies are individually validated for chromatin precipitation, every lot, every time." We also found robust binding at REST ChIP-seq peaks, and no significant binding at control sites situated 10 kb downstream (Fig. 4d,e), further confirming the specificity of this antibody.

The REST14 antibody, which we used to label mouse REST by immunofluorescence (gift from Jenny Hsieh), has been validated for immunofluorescent labeling of mouse REST (see Mukherjee et al. Nat. Communications 7, 13360 (2016)). We further validated this antibody using WT and REST KO tissue, as follows: (1) using WT and brain-specific REST conditional KO (Nestin-Cre;REST (lx/lx)) tissue, we found an almost complete loss of immunofluorescence signal in REST conditional KO tissue (see Zullo et al. Nature 574, 359-364 (2019)). (2) in WT mice with a glutamatergic-specific REST KO (CamKlla-Cre; REST(lx/lx)), we found loss of REST immunofluorescence in most neurons of forebrain (see Supplementary Fig. 5a). (3) in 3xTg mice with a glutamatergic-specific REST KO (3xTg; CamKlla-Cre; REST(lx/lx)), we also found loss of REST immunofluorescence in forebrain neurons (see Supplementary Fig. 5e).

The tau antibodies obtained from Peter Davies against pSer202 tau (CP13 clone), conformationally altered tau (MC1 clone) and pSer396 tau (PHF1) have been extensively validated and cited in the field. We also found that PHF1 recognizes tau species at the expected molecular weight in western blotting (Fig 7d).

The mouse monoclonal antibody that recognizes phosphorylated Ser202/Thr205 tau (clone AT8; ThermoFisher Scientific, Catalog No. MN1020) underwent advanced verification by ThermoFisher Scientific to ensure it binds the antigen stated. The antibody has been cited 817 times, and validated for use in western blotting. We also found that it recognizes pSer202/Thr205 tau in western blotting at the expected molecular weight (data not shown).

The mouse monoclonal antibody that recognizes phosphorylated Thr231 tau (clone AT180; ThermoFisher Scientific, Catalog No. MN1040) has been cited 817 times, and validated for use in western blotting. We also found that it recognizes pThr231 tau in western blotting at the expected molecular weight (Fig. 7d, Supplementary Fig. 8a).

The rabbit polyclonal antibody that recognizes phosphorylated Thr217 tau (ThermoFisher Scientific, Catalog No. 44-744) has been cited 20 times, and validated for use in western blotting. We also found that it recognizes pThr217 tau in western blotting at the expected molecular weight (Supplementary Figure 8a).

The mouse monoclonal antibody that recognizes all tau species (total tau; clone tau-5; ThermoFisher Scientific, Catalog No. AHB0042) underwent advanced verification by ThermoFisher Scientific to ensure it binds the antigen stated. The antibody has been cited 97 times, and validated for use in western blotting. We also found that it recognizes tau in western blotting at the expected molecular weight (Fig. 7d, Supplementary Fig. 8a).

The anti-human Aβ rabbit monoclonal IgG antibody (Cell Signaling, Cat. No. 8243) has been cited 112 times, and validated for use in IF, as well as WB and IP. We also found that it recognizes oligomeric amyloid beta in young J20 but not WT mice (Suppl Fig. 2c) and labels amyloid plaques in J20 and 3xTg mice (Fig. 2i, 7f, 9h, Suppl Fig. 7d, 7h).

The anti-human APP mouse monoclonal IgG1,K antibody (clone 6E10; Biolegend, Catalog No. 803001) has been cited 383 times, and validated for use in WB (quality tested by Biolegend), ELISA and IHC. We also found that it recognizes human APP in western blotting at the expected molecular weight (Supplementary Fig. 5h,k).

The anti-actin mouse monoclonal IgG1 antibody (clone ACTN05 (C4); ThermoFisher Scientific, Catalog No. MA5-11869) has been cited 340 times, and validated for use in WB, IHC and IP. We also found that it recognizes actin in western blotting at the expected molecular weight (Fig. 5i, 7d, Supplementary Fig. 5c, 8a).

The anti-NeuN mouse monoclonal IgG antibody (clone A60, Millipore, MAB377) is "routinely evaluated by immunohistochemistry on brain tissue" (Millipore). We also found that it recognizes the nuclear NeuN antigen in IHC (Suppl Fig. 9e).

The anti-MAP2 goat polyclonal IgG antibody (PhosphoSolutions, Catalog. No. 1099-MAP2) is a protein G-purified antibody that has been validated by PhosphoSolutions for use in IHC/IF and WB. We also found that it recognizes the cytosolic MAP2 antigen in neurons in human and mouse tissue (Fig. 5a,c,e,g and Suppl Fig. 1d, 6a,d,g).

The anti-CDK5 mouse monoclonal IgG antibody (clone 4E4; Novus Bio, Catalog No. NBP2-37602) has been cited by 1 previous paper, and validated for use in WB, ICC/IF, IHC, ELISA, and flow cytometry. We also found that the antibody recognizes a single major CDK5 band in WB (Fig 5i).

The anti-GSK3β mouse monoclonal IgG antibody (clone D5C5Z; Novus Bio, catalog No. NBP1-47470S) been cited 8 times, and validated for use in WB, ICC/IF, IHC, ELISA, and flow cytometry. We also found that the antibody recognizes a single major GSK3β band in WB (Fig 5i).

The anti-PS1 C-terminal (CTF) rabbit monoclonal IgG antibody (clone EP2000Y; Abcam, Catalog No. ab76083) been cited 37 times, and validated for use in WB and IHC, as well as knockout-validated by Abcam. We also found that the antibody recognizes PS1 in WB at the expected molecular weight (Fig 6e).

The anti-PS1 N-terminal (NTF) rabbit polyclonal IgG antibody (231-f; made in the Yankner lab) has been validated in the Yankner laboratory in WB and has been used and cited by other laboratories. We also found that the antibody recognizes PS1 in WB at the expected molecular weight (Fig 6e).

The anti-Nicastrin mouse monoclonal IgG antibody (clone 9C3; Biolegend, Catalog No. 852301) has been cited by 1 previous paper, and validated for use in WB and IHC-P by Biolegend. In addition, "each lot of this antibody is quality control tested by Western blotting" (Biolegend).

The anti-Nicastrin rabbit polyclonal IgG antibody (Sigma Millipore, Catalog No. N1660); anti-PEN2 rabbit polyclonal IgG antibody

(ProScience, Catalog No.3981) has been cited 96 times, and is an "enhanced validation antibody" validated by Signa-Aldrich for use in multiple type of assays (WB, IHC, ICC, ELISA). We also found that the antibody recognizes Nicastrin in WB at the expected molecular weight (Fig 6e).

The anti-PEN2 rabbit monoclonal IgG antibody (clone EPR9200; Abcam, Catalog No. ab154830) been cited 5 times, and validated for use in WB, IHC and flow cytometry, as well as knockout-validated by Abcam. We also found that the antibody recognizes PEN2 in WB at the expected molecular weight (Fig 6e).

The anti-PEN2 rabbit polyclonal IgG (ProScience, Catalog No. 3981) has been cited by 2 previous papers, and validated for use in WB, IHC-P, IF and ELISA by ProScience.

The anti-Transferrin receptor mouse monoclonal IgG antibody (clone H68.4; ThermoFisher Scientific, Catalog No. 13-6800) been cited 520 times, and underwent "advanced verification" by knockdown (ThermoFisher Scientific). It is validated for use in WB, ICC/IF, IHC, ELISA, IP and flow cytometry. We also found that the antibody recognizes a single major band in WB (Fig 6e,g).

The anti-BiP/GRP78 mouse monoclonal IgG (clone C38; ThermoFisher Scientific, clone C38, Catalog No. 14-9768-82) been cited 3 times, and has been validated for use in IHC-P, ICC/IF and ELISA by ThermoFisher Scientific.

The anti-β-catenin goat polyclonal IgG (R&D Systems, Catalog No. AF1329) has been cited 20 times, and has been validated for use in WB, IHC, ICC and flow cytometry by R&D Systems.

The non-specific rabbit IgG antibody (Sigma Millipore, Catalog No. 17-641) has been confirmed to serve as a non-specific rabbit IgG control in WB, IP, ChIP and ChIP-seq by Sigma Millipore. We have also used this control antibody for ChIP-PCR (Fig. 4e).

The anti-FLAG mouse monoclonal IgG1 antibody (clone M2; Sigma Millipore, Catalog No. F3165) been cited 7,833 times and is validated for use in WB.

Eukaryotic cell lines

Policy information about cell lines and Sex and Gender in Research

Cell line source(s)	The SH-SY5Y cells were obtained from the American Type Culture Collection (ATCC). The WT and REST KO mouse embryonic fibroblasts (MEF) cell lines were established in our laboratory using pooled embryonic fibroblasts from both male and female embryos (1:1 ratio) (see Methods).
Authentication	The identity of the SH-SY5Y cell line was verified by the supplier, The American Type Culture Collection (ATCC). In addition, cell line authentication was performed based on its characteristic morphology. The MEF cell lines were generated in the Yankner lab, using an established protocol. The genotypes of WT and REST KO MEFs were confirmed by PCR (see Figure 6b). Loss of REST in KO MEFs has been confirmed by both mRNA and protein analysis (see Figure 6b,c,d).
Mycoplasma contamination	Not tested.
Commonly misidentified lines (See ICLAC register)	No commonly misidentified cell lines were used in this study.

Animals and other research organisms

Policy information about studies involving animals; ARRIVE guidelines recommended for reporting animal research, and Sex and Gender in Research

Laboratory animals

All mice were housed socially (2-4 animals/cage) in a room with a 12-hour light/dark cycle (lights on at 6:00 am), controlled for temperature (18-22°C) and humidity (40-60%). Sentinel mice housed in each rack were tested quarterly and confirmed free of pathogens.

Mice carrying floxed alleles of REST flanking exon 2 were obtained from William Klein (University of Texas MD Anderson Center) (described by Mao et al. Dev Biol 349, 90-9 (2011)). CA1-specific CamKIIα-Cre (Tsien et al. Cell 87, 1317-26 (1996)) or forebrain-specific CamKIIα-Cre mice (Yu et al. Neuron 31, 713-26 (2001)) were obtained from Jie Shen (Harvard Medical School), and employed to achieve REST conditional inactivation in hippocampus CA1 pyramidal neurons, or cortical and hippocampal excitatory glutamatergic neurons, respectively. Mice carrying a REST genetrap (REST(GT)) null allele were obtained from Wolfgang Wurst and Thomas Floss (Nechiporuk et al. Elife 5, e09584 (2016)). REST(GT/+) mice are viable and have been maintained as long as 29 months. The CamKIIα-Cre transgenes are in the C57BL/6J background, and the RESTlx/lx alleles is in a hybrid C57BL/6J and 129Sv/Ev background. The REST GT allele was in a C57BL/6J background. The J20 transgenic mice (Mucke et al. J Neurosci 20, 4050-8 (2000)) express a mutant form of the human amyloid protein precursor bearing both the Swedish (K670N/M671L) and the Indiana (V717F) mutations (APPSwlnd) in a C57BL/6J backgroundll. The 3xTg mice (Oddo et al. Neuron 39, 409-21 (2003)) carry APPSwe and tauP301L mutant transgenes, as well as a PS1 knock-in mutation and were in a hybrid C57BL/6J and 129Sv/Ev background. The J20 and 3xTg lines were obtained from The Jackson Laboratory.

To generate mutant mice, we typically set up 10-20 females (all littermates derived from the same cross) with 8-12 males (all littermates derived from the same cross). CamKIIα-Cre:RESTlx/lx conditional knockout mice, or controls (RESTlx/lx, RESTlx/+ or CamKIIα-Cre) in either WT, J20 or 3xTg backgrounds (hybrid C57BL/6J and 129Sv/Ev background), as well as RESTGT/+ or REST+/+ mice, in either WT, J20 or 3xTg backgrounds (hybrid C57BL/6J and 129Sv/Ev background) were born at expected Mendelian ratios, were viable and fertile, and did not display any visible alterations.

Mice were identified by numbered ear tags and were randomly selected for behavioral studies and histological analyses.

To determine whether REST is involved in the onset and progression of AD pathology and cognitive decline, we examined AD mice (3xTg and J20 models) carrying REST knockout or WT alleles. To determine whether genetic deletion of REST enhances AD pathology, we employed animals with a mild AD phenotype, which thus allow the detection of any enhancement of pathology and cognitive function upon REST deletion. Hemizygous 3xTg mice display a mild accumulation of amyloid and tau, and were employed for genetic enhancement studies. These mice were crossed to mice carrying Cre and floxed REST alleles, or mice carrying the REST gene trap allele.

Once the desired genotypes were obtained, mice were subjected to the aging process. Mice were analyzed at defined periods during the aging process. For crosses involving 3xTg, mice were analyzed at 17-18 or 28 months of age, given the milder phenotype of the 3xTg hemizygous mice. Mice with a complete REST deletion in forebrain excitatory neurons (3xTg carrying floxed REST alleles and CamKIIα-Cre) were analyzed at 17-18 months. Mice with a partial (heterozygous) REST deletion (3xTg carrying the REST gene trap allele) were aged to ~28 months (27.7 ± 0.9 months for 3xTg and 27.5 ± 0.7 months for 3xTg;GT).

The J20 mice display a more robust accumulation of amyloid, with onset of plaque formation around 6 months of age. The J20 mice with a complete conditional REST deletion in neurons (J20;cKO) were analyzed at 12-14 months of age (13.1 ± 0.7 for J20; 13.2 ± 0.8 for J20;cHET and 13.2 ± 0.6 for J20;cKO), whereas J20 mice with a partial, heterozygous, REST gene trap deletion (J20;GT) were analyzed at 27-29 months of age (28.1 ± 1 months for J-20 and 27.9 ± 1 months for J-20;GT mice).

Most experiments included both male and female mice (information about the gender of all animals used in the various experimental groups is available in the Supplementary Data 1). Unless otherwise stated, gender had no significant effect on the measured dependent variables, nor did it significantly interact with genotype to influence the measured variables (see Supplementary Data 1 for two-way ANOVA analyses of gender and genotype effects, as well as gender x genotype interactions).

Wild animals — No wild animals were used in this study.

Reporting on sex — Both males and females were included in the study. The influence of gender on the measured variables was assessed if the experimental groups comprised both males and females (see Supplementary Data 1).

Field-collected samples — No field-collected samples were used in this study.

Ethics oversight — Animal housing and experimental procedures were approved by the Institutional Animal Care and Use Committee of Harvard Medical School.

Note that full information on the approval of the study protocol must also be provided in the manuscript.

ChIP-seq

Data deposition

☒ Confirm that both raw and final processed data have been deposited in a public database such as GEO.

☒ Confirm that you have deposited or provided access to graph files (e.g. BED files) for the called peaks.

Data access links
May remain private before publication. — ChIP-seq data has been deposited at Gene Expression Omnibus (GEO): ncbi.nlm.nih.gov/geo/query/acc.cgi?acc=GSE195446

Files in database submission —
REST_ChIP-seq_WT_rep1
REST_ChIP-seq_WT_rep2
REST_ChIP-seq_WT_rep3
REST_ChIP-seq_WT_rep4
REST_ChIP-seq_3xTg_rep1
REST_ChIP-seq_3xTg_rep2
REST_ChIP-seq_3xTg_rep3
REST_ChIP-seq_3xTg_rep4
Input-WT
Input-3xTg

Genome browser session
(e.g. UCSC) —
Platform: GPL19057
Series: GSE195446

Methodology

Replicates — Four biological replicates, each comprised of n=4 pooled frozen cortices from 3xTg mice (females, age 11 months) were processed for ChIP-seq (total n=16 3xTg cortices). Similarly, four biological replicates, each comprised of n=4 pooled frozen cortices from WT mice (females, age 11 months) were processed for ChIP-seq (total n=16 WT cortices).

Sequencing depth — Illumina sequencing libraries were prepared from the ChIP and Input DNAs by the standard consecutive enzymatic steps of end-polishing, dA-addition, and adaptor ligation. Steps were performed on an automated system (Apollo 342, Wafergen Biosystems/Takara). After a final PCR amplification step, the resulting DNA libraries were quantified and sequenced on Illumina's NextSeq 500 (75 nt reads, single end).

Antibodies — For ChIP-seq, we used a REST C-terminal antibody (generous gift from Gail Mandel, Vollum Institute) (McGann et al. J. Neurosci 41,

Antibodies	6582-6595 (2021); Ballas et al. Cell 121, 645-657 (2005)).
Peak calling parameters	Reads were aligned to the mouse genome (mm10) using the STAR algorithm (v2.7.0a) (--alignIntronMax 1 --alignEndsType EndToEnd). Peak locations were determined using the MACS algorithm (v2.1.0) (--nomodel) and normalized fold enrichment tracks were generated by using the callpeak function with --SPMR, then passing the bedgraph outputs into the bdgcmp function with the setting -m FE (fold enrichment). Bedgraph files were converted into bigWig and visualized using Integrated Genome Viewer (IGV, v2.4). Peaks were filtered for fold enrichment more than 5 and q-value less than 0.01.
Data quality	Peaks that were on the ENCODE blacklist of known false ChIP-seq peaks were removed. See Supplementary Table 2 for a summary of the REST ChIP-seq experiment.
Software	Reads were aligned to the mouse genome (mm10) using the STAR algorithm(v2.7.0a). Peak locations were determined using the MACS algorithm (v2.1.0) (--nomodel) and normalized fold enrichment tracks were generated by using the callpeak function with --SPMR, then passing the bedgraph outputs into the bdgcmp function with the setting -m FE (fold enrichment). Bedgraph files were converted into bigWig and visualized using Integrated Genome Viewer (IGV, v2.4). Motif enrichment analysis was performed using HOMER. Bedtools Intersect was used to overlap peaks from WT and 3xTg samples to determine common regions in each group. Differential binding sites was identified using DiffBind (https://bioconductor.org/packages/devel/bioc/vignettes/DiffBind/inst/doc/DiffBind.pdf) using default settings. Peaks were annotated using ChIPseeker, computeMatrix (v3.3.2) was applied to build a matrix with a flanking region of ± 1 kb around the center of enriched REST peaks and plotHeatmap (v3.3.2) was used to generate heatmaps. We employed DAVID for functional enrichment analysis and enrichment of gene ontology (GO) terms.
